# Application of single-cell RNA sequencing on human testicular samples: a comprehensive review

**DOI:** 10.7150/ijbs.82191

**Published:** 2023-04-09

**Authors:** Fan Dong, Ping Ping, Yi Ma, Xiang-Feng Chen

**Affiliations:** 1 Center for Reproductive Medicine, Ren Ji Hospital, School of Medicine, Shanghai Jiao Tong University, Shanghai, China.; 2 Shanghai Key Laboratory for Assisted Reproduction and Reproductive Genetics, Shanghai, China.; 3 Shanghai Human Sperm Bank, Shanghai, China.

**Keywords:** single-cell RNA sequencing, human testis, male gonad, spermatogenesis, bioinformatic analysis

## Abstract

So far there has been no comprehensive review using systematic literature search strategies to show the application of single-cell RNA sequencing (scRNA-seq) in the human testis of the whole life cycle (from embryos to aging males). Here, we summarized the application of scRNA-seq analyses on various human testicular biological samples. A systematic search was conducted in PubMed and Gene Expression Omnibus (GEO), focusing on English researches published after 2009. Articles related to GEO data-series were also retrieved in PubMed or BioRxiv. 81 full-length studies were finally included in the review. ScRNA-seq has been widely used on different human testicular samples with various library strategies, and new cell subtypes such as State 0 spermatogonial stem cells (SSC) and stage_a/b/c Sertoli cells (SC) were identified. For the development of normal testes, scRNA-seq-based evidence showed dynamic transcriptional changes of both germ cells and somatic cells from embryos to adults. And dysregulated metabolic signaling or hedgehog signaling were revealed by scRNA-seq in aged SC or Leydig cells (LC), respectively. For infertile males, scRNA-seq studies revealed profound changes of testes, such as the increased proportion of immature SC/LC of Klinefelter syndrome, the somatic immaturity and altered germline autophagy of patients with non-obstructive azoospermia, and the repressed differentiation of SSC in trans-females receiving testosterone inhibition therapy. Besides, the re-analyzing of public scRNA-seq data made further discoveries such as the potential vulnerability of testicular SARS-CoV-2 infection, and both evolutionary conservatism and divergence among species. ScRNA-seq analyses would unveil mechanisms of testes' development and changes so as to help developing novel treatments for male infertility.

## Background

Human testis is the core organ of the male reproductive system 1, and acts as the cornerstone of male fertility since it is the place of spermatogenesis 2. On the one hand, the testis is lionized by developmental biologists because it's the start of sex determination 3 and the kindergarten of male germline cells 4. Meanwhile, human testis is also the priority of andrologists because testicular failure is the leading cause of male infertility 4. Therefore, scientists have delved into both normal 5, 6 and abnormal testes 7-9, and detected mechanisms behind them especially those related to male infertility 10, 11. Although numerous approaches have been used to learn about human testis including cell experiments 12, animal experiments 13 or even new methods like testicular organoid 14, most of the above-mentioned methodologies are indirect. It is no doubt that the most ideal model to study human testes is the testis itself. Therefore, human testicular biological sample-based studies could be important sources of knowledge of changes in both normal and impaired testes.

As early as the 1910s, human sample-based studies had started to give direct evidence of testicular diseases 15. Yet until the 1970s, most of those studies were restricted to anatomy and morphology/histology 16-19 perhaps because at that time nonspecific histological staining was the most popular way to study human testes. Then with the development of some more specific methods such as immunohistochemistry staining (IHC), researches on the human testis reached protein levels 20-22. A huge breakthrough in this field was the application of microarray and high-throughput RNA-seq on human testicular samples 23-26, which gave scientists a transcriptional view of both the normal testis's development and the pathogenesis of spermatogenic dysfunctions 27, 28. Nevertheless, both microarray and traditional RNA-seq are based on bulk tissue, for which the testicular sample is considered as a whole, so we could only obtain the mainstream of transcriptional changes in the sample but ignore the heterogeneity of different cells. To solve this problem, Tang et al. introduced single-cell RNA sequencing (scRNA-seq) 29, which could detect the transcriptome at a single-cell resolution. The technology was created on blastomere and oocytes and soon applied to human testicular cells 30 and then to unsorted testicular tissues 31. So far, there have been plenty of studies using human testicular scRNA-seq data and several reviews partially or roughly touched upon this topic (human testicular scRNA-seq analysis) 32-39. Nevertheless, some of these reviews didn't fully focus on “human testis” and/or “scRNA-seq” (for example, some included much more about female gonad or animal testis 32, 34, 40 and some focused much on techniques other than scRNA-seq 33, 37, 38), which led to limited paragraphs and blurry outline on this topic. Other reviews were almost restricted to germ cells 35, 36 or after-birth gonads 39, which gave an incomplete depiction of this topic. Most importantly, none of these articles was with systematic literature searching strategies, which might result in a biased interpretation. Using systematic literature searching, this comprehensive review plans to fully summarize the new findings offered by scRNA-seq analyses of human testicular biological samples, concerning both testicular cells and unsorted testicular tissues, both prenatal and postnatal male gonads, both underage and adult testes, both young and aged testes, both normal spermatogenic and impaired spermatogenic patients, and both original sequencing data and reusage of published data. We hope this review will give scientists detailed and all-round information on testicular development, spermatogenesis as well as testicular pathogenesis in the single-cell resolution, and humbly suggest the future directions in this field.

## Methods

### Search methods

The searches were conducted on 29 Aug 2022. First, a systematic search in PubMed database was conducted. Based on the fact that this review mainly focused on two aspects, including “scRNA” and “human testicular samples”, we used a matched searching strategy to link these two aspects. In detail, for the former aspect, the terms “single-cell” (term A) or “scRNA” (term B) were selected. And each of these two terms was combined with one of the following testis-related (the latter aspect-related) search terms, including “testis” (term 1), “spermatogenesis” (term 2), “Klinefelter Syndrome” (term 3), “azoospermia” (term 4), “Y chromosome microdeletion” (term 5), “cryptozoospermia” (term 6), “oligozoospermia” (term 7), “asthenospermia” (term 8), “teratospermia” (term 9), “orchitis” (term 10), “cryptorchidism” (term 11) and “male and gonad” (term 12). In order to minimize the chance of omitting related studies especially preprint studies, we do an extra search on GEO dataset (https://www.ncbi.nlm.nih.gov/gds) using four combination of advanced search terms including “((testis) AND scRNA) AND Homo sapiens [Organism]”, “((testis) AND single-cell) AND Homo sapiens[Organism]”, “(((male) AND gonad) AND scRNA) AND Homo sapiens[Organism]” and “(((male) AND gonad) AND single-cell) AND Homo sapiens[Organism]”, which also referred to the data related to both focused aspects. For filtering, the “series” of Entry type and “Expression profiling by high throughput sequencing” of Study type were also chosen. For each identified GEO series, its related articles were obtained from GEO or bioRxiv (https://www.biorxiv.org/) if there was no published article at the searching time.

### Selection criteria

The inclusion criteria were (1) studies with single-cell RNA sequencing applied on human testicular biological samples (including testicular tissues, cells and prenatal male gonads) and/or (2) studies with re-analyses of published/public scRNA-seq data of human testicular biological samples (not including pure literature review of published articles).The exclusion criteria were (1) articles not in English; (2) articles published before 2009 (the year when scRNA-seq technology was created 41); (3) non-research articles (such as pure review/case report/guideline/protocol/technical note/correction/comment/chapter of book/letter) without scRNA-seq analysis on human testis; (4) full-length articles not available; (5) tumor studies without scRNA-seq data analyzing or not relevant to human testis; (6) studies based on sequencing methods other than scRNA-seq (e.g. single-cell small RNA-seq or spatial transcriptomics sequencing); (7) pure animal or botanic studies; (8) human-related studies but with no scRNA-seq analysis on human testicular samples.

## Results

### Application of scRNA-seq on human testicular samples: an overview

**Figure 1:**
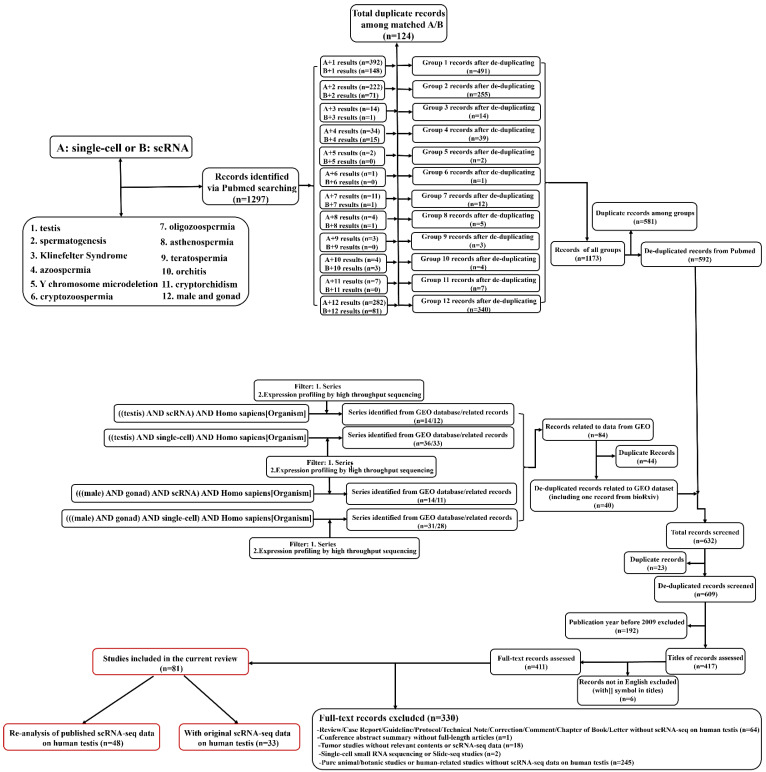
Workflow of the literature searching and study selection

The workflow of literature searching and selections was in **Figure 1**. The searches returned about 1300 records (609 after de-duplication). By further filtering, 81 full-length articles were enrolled into the final analysis. There were 33 articles containing original scRNA-seq data (at least from one donor) on human testicular samples, while the rest 48 articles were based on the re-analysis of previously published data (no matter whether the data was previously published by the same team). Since the year of 2015, Guo et al. for the first time do scRNA-seq on human fetal testicular cells 30, followed by adult testicular cells sequenced by two studies in 2017^42^, ^43^. Interestingly, although those two adult studies were from different teams, both of them focused on spermatogonia of one accord. From 2018, numerous studies have done scRNA-seq on human testicular samples regarding both prenatal and postnatal, both normal and abnormal testes. **Table 1** summarized articles that have originally published scRNA-seq data on human testicular samples. We could conclude that most of the studies were conducted by scientists from the USA and China. As for library techniques, most of the studies used the 10×Genomics platform (https://www.10xgenomics.com/) to carry out their works. While other studies chose techniques/plateforms such as BD Rhapsody 44, (modified) smart-seq2 45-47, STRT-seq 48, Microwell-seq 49, Drop-seq 50, inDrop 51, Slingleron GEXSCOPE^®^ 52, 53 and some earlier platform such as Fluidigm C1 54 and Tang method 29, 41. In some studies 55, 56, two or more methods simultaneously appeared for cell capturing. So far, no comparative study has focused on which technique is more suitable for scRNA-seq of human testicular samples. Nevertheless, some reviews and comparative studies based on scRNA-seq of other tissues have gradually revealed that the technological differences of scRNA-seq, including both the wet-lab parts (e.g., cell capturing/sequencing/barcoding) 44, 57-59 and the dry-lab parts (e.g., data-processing/batch-effect correction/dimension reduction/quality control) 60-64 might have profound impact on the final results. Especially, unavoidable technological batch effects will be generated using data generated from different sequencing platforms 65. Although the data will go through batch effects correction before downstream analysis 60, we still recommend that researchers should choose one suitable techniques and consistent data-analyzing methods to do their testicular scRNA-seq works. If you need to do integrated analyses and have to combine your own scRNA-seq data with public scRNA-seq data, we suggest that you should choose the public data that used the same cell-capturing techniques as your own data, to minimize the non-biological batch effects.

In terms of sample types, one-third of the studies choose prenatal male gonads (the youngest sample was from the gonad from a 4-week male embryo). For adult testes (the oldest sample was from a 76-year-old male for now), most of the studies included samples with complete/full/normal spermatogenesis. But the sources of these normal spermatogenic samples varied. Some collected deceased healthy male testicular samples 31 to serve as the normal groups, which were ideal controls. For many institutes, obstructive azoospermia (OA) samples with full spermatogenesis confirmed by histopathological diagnosis, including congenital bilateral absence of the vas deferens 66, post-vasectomy 67, and physical obstruction of the vas deferens 66 were considered as workarounds for normal/control groups. It is noteworthy that in the original manuscripts of Hermann et al. 68, Tan et al. 69 and Sohni et al 67, some of the adult testicular biopsies were obtained from patients receiving vasectomy reversal, and the samples in Xia et al.'s work 70 were from testicular sperm extraction (TESE) surgeries of patients with obstructive infertility (they were also post-vasectomy patients after confirming with the first author). Interestingly, none of these patients was clearly claimed as OA patients, although they all had obvious obstructive etiology. Frankly speaking, these post-vasectomy patients were extremely unlikely to have sperm in ejaculate (only if some rare situations happened such as duplication of vas deferens or natural recanalization 71). But in order to be strict and consistent, in the current review, all patients with obstructive etiology (no matter they were diagnosed with obstructive azoospermia or not) were also unitedly abbreviated as obstruction (OB). Besides, testes removed from patients receiving transsexual operation might also be an option for sample collection 43, 72. Additionally, normal testicular samples at age 17 were collected by both Zhao's and Guo's studies, but were assigned to different groups (adult for Guo's and underage for Zhao's studies) 31, 55, maybe due to the different definition of adult (biological adult versus legal adult).

**Figure 2:**
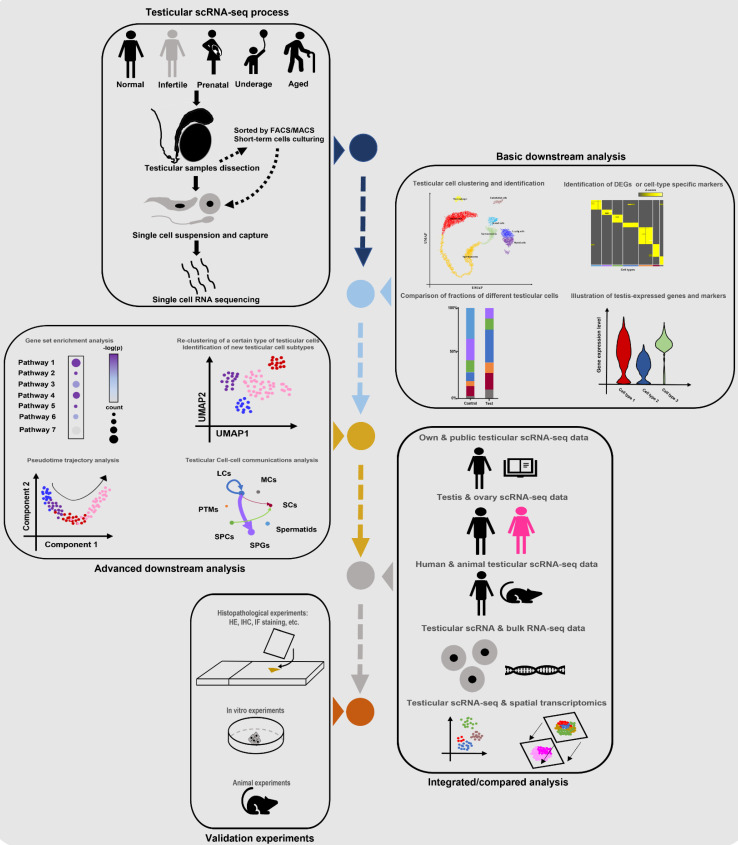
Standard guideline/workflow of single-cell RNA sequencing studies on human testicular biological samples. Some of the elements used in this figure were downloaded from Icons of Microsoft PowerPoint 2021(Microsoft, USA). Note: scRNA-seq, single-cell RNA sequencing; FACS, fluorescence activated cell sorting; MACs, magnetic activated cell sorting; DEGs, differentially expressed genes; HE, hematoxylin-eosin; IHC, immunohistochemistry; IF, immunofluorescence.

Till now, the number of scRNA-seq testicular samples in each study has been limited, maybe due to the high price of scRNA-seq commercial service and limited sample donors. To partially solve this dilemma, some studies chose to do combined analyses of their own scRNA-seq data and public scRNA data or bulk RNA-seq data. And most of the studies in **Table 1** did extra experiments (such as histological experiments, animal or cell experiments) to validate the findings from scRNA-seq analyses. Additionally, some studies were conducted on both testes and ovaries 22, 73, or on testes from both human and animals 68, in order to do comparisons between genders or among species. Garcia-Alonso et al did integrated analyses by the combination of testicular scRNA-seq with spatial transcriptomics 74, which is a novel direction. Nevertheless, so far there has been no guideline for scRNA-seq studies on the human testis. By reviewing all related studies, we summarized a standard guideline(workflow) of testicular scRNA-seq-based studies (**Figure 2**). We believe that with the wider application of scRNA-seq techniques, there will be more scRNA-seq-based studies of testicular samples published, with more comprehensive analyzing methods used.

### Cell identifications by scRNA-seq on human testicular samples

The fundament of scRNA-seq analysis is cell identification. Apart from a few studies based on isolated cells by fluorescence activated cell sorting (FACS) or magnetic activated cell sorting (MACS), the majority of studies in **Table 1** were based on testicular tissues. For adult testes, the representatively identified cell types in scRNA-seq analyses were summarized in **Table 2**. ScRNA-seq-identified testicular cells could be roughly divided into three classifications, including spermatogenic cells (germ cells), non-immune somatic cells and immune cells 66. The widely used and some newly identified markers for cell identification were also summarized (**Table 2**). Importantly, because of the different study purposes and different sample selections, the types and subtypes of testicular cells in different studies were slightly variant. For germ cells, spermatogonia (SPG), spermatocyte (SPC) and spermatid could be broadly identified in a full-spermatogenic sample if the focus of the study is not spermatogenic cells 55. Nevertheless, a more detailed classification of these three germ cells could be done based on stage-specific markers. Here we recommended a moderate classification of germ cells used by Nie et al., in which SPG could be further divided into spermatogonial stem cell (SSC) (also called undifferentiated SPG) and differentiating SPG (diff_SPG), SPC into early primary SPC and late primary SPC, spermatid into round and elongating/elongated spermatid 75. One can adjust the subdivision of each germ cell type according to your research focus. For example, if you were not interested in the development of SPC, you could easily keep SPC as an unclassified type but classifying SPG and spermatid into more detailed subtypes 56. If the whole spermatogenesis is your attention, you might choose a more aggressive strategy for germ cell classification 66, 76.

Apart from germ cells, it is well known that somatic cells also played important roles in spermatogenesis. Such somatic cells could be further divided into non-immune somatic cells and testicular immune cells. For non-immune somatic cells, Sertoli cells (SC) were one of the most important cell types in spermatogenesis, as they could not only form blood-testis barrier but also directly support germ cell survival 55. And Leydig cells (LC) could affect spermatogenesis by producing testosterone 75. Although the roles of testicular immune cells in spermatogenesis have not been fully revealed, more evidence indicated that they could interact with germ cells and take part in spermatogenesis 77.

The rules of cell identification in germ cells also worked when annotating somatic cells. For example, although the rest studies all keep testicular macrophages as a whole, Mahyari et al. chose to further divided them into M1 and M2 macrophages 78. Nevertheless, the above-mentioned all classifications were “well-known” classification of adult testicular cells. One important function of scRNA-seq is to identify novel subtypes of cells. For example, traditionally SC were considered as one type of cells, but Zhao et al. and Guo et al. found three subtypes of SC in the human testis 55, 72. Similar findings also occurred in LC 78. Owing to the lack of unified understanding, sometimes similar novel subtypes of certain cells were independently reported by different teams with different nomenclatures. For instance, an important subtype of prenatal gonadal cells which could bifurcate into the developmental path of both SC and LC were reported by two different teams with different names 79, 80. Meanwhile, samples with different pathological status might deeply influence the identified cell types and numbers. A case in point is testicular immune cells. In the microenvironment of testes with spermatogenic dysfunction, an increasing number of immune cells were observed 66. Additionally, the main type of immune cells in full spermatogenic testes was macrophages, while a large number of mast cells were identified in testes from non-obstructive azoospermia (NOA) patients, especially in Sertoli cell-only syndrome (SCOS) 55, 56.

### ScRNA-seq demonstrated the development and changes of testicular cells from all ages

#### Testicular development from prenatal to postnatal period

##### 1. Overview

Before the year 2015 in which the first prenatal testicular sample was scRNA-seqed, the understanding of the development of different types of testicular cells in prenatal testes was limited to the changes of cell number, morphology and distribution 81. So far, with over ten articles published their own scRNA-seq data on human prenatal testicular samples, we already have an outline of both germline and somatic development at single-cell transcriptional level.

##### 2. Germline transition

For germline development pre- and postnatally, Guo et al pointed out that prenatal germs cells generally have two main forms of transcriptional profiling, including a form of primordial germ cells (PGC) (expressing PGC markers such as *TFAP2C*, *KIT*, *NANOG*, *POUF51* and *SOX17*) and form of the most naïve SSC (expressing earliest SSC markers such as *PIWIL4*, *EGR4*, *MSL3* and *TSPAN33*) 79. PGC form mainly started at the embryotic time and then gradually stopped meiosis and inhibited pluripotency and at transcriptional level became cells with high similarity to the early status of adult SSC 79. The latter form (named State f0) 79 strode across human birth and kept a quite similar transcriptional status until the adult time (named State 0 in adult) 31. During the germline transition from PGC to (f)State 0, pathways related to gonad and stem cell development were downregulated while signaling related to transcription/homeobox were enhanced 79. Similarly, Wang et al. found a transition from mitotic (mainly existing before 15 weeks) to meiotic arrest (mainly existing after 15 weeks) male fetal germ cells (FGC) 82, which was in line with Guo's 79 and Li's results 73, indicating that the downward status of meiosis (proliferative-quiescent-arrested) was the symbol of male fetal germ cell development. Garcia-Alonso et al. called the latter form of FGC prespermatogonia and also showed the activation of EGR4 in this stage 74. Interestingly, Wang et al. also reported an extra subtype of FGC called SPARC^+^ POU5F1^+^ FGC, which might keep the ability to migrate locally within the fetal testis 82.

##### 3. Somatic development

As for prenatal somatic cells, most of the researches focused on SC and LC due to the isogenesis of these two cells. The source of testicular somatic cells was embryotic coelomic epithelium (CE) 83. By re-clustering of scRNA-seqed CE cells, Cheng et al. found two subclusters of CE, of which one was *NR0B1*^+^*STAR*^+^*NR5A1*^+^ but *GATA4*^-^*LHX9*^-^ (annotated as adrenogenic CE), while the other was *GATA4*^+^*LHX9*^+^*HOXA9*^+^*HOXD9*^+^ but *NR5A1*^-^ (known as posterior/gonadogenic CE) 74, 80. The former subcluster will develop into the adrenal gland while the latter goes to the testis. Noteworthily, gonadogenic CE will step into a sort of bipotent somatic progenitors (symbolized by the expression of *NR5A1*), which had been recognized by several studies 79, 80. In Wang et al.'s study, a subpopulation of KRT19^+^ somatic cells were identified mainly in 6- to 8-week testes, which also resembled these somatic progenitors 82. Starting from 7-8 weeks, these somatic progenitors bifurcated into two different developmental paths, one of which is embryonic Sertoli progenitors (marked by* SRY*, similar to the “early supporting gonadal cells” in Garcia-Alonso et al.'s work 74) and then became fetal SC 79. The rest path, on the contrary, led to embryonic/fetal interstitial progenitors (marked by *ARX* and *TCF21*, also called Leydig precursor cells by Li et al. 73 and called somatic progenitor by Wang et al. 84), and finally part of the fetal interstitial progenitors became fetal Leydig cells (marked by *HSD3B2* and *CYP17A1*, also called differentiated LC by Li et al. 73) 79, 80. During the development of prenatal interstitial/Leydig lineage, the enriched molecular pathways altered from “extracellular matrix”/ “cell adhesion” to “steroid biosynthesis”. Interestingly, at the perinatal stage, fetal SC successfully passed the birth (with genes related to translation and respiratory chain enhanced, while genes related to the endoplasmic reticulum and steroid biosynthesis reduced) and became postnatal SC including stage_a and b (discussed in more detail below) 55, 79, while fetal Leydig cells disappeared in the postnatal time 79. In fact, it was CYP17A1-negative fetal interstitial cells that might finally pass the birth and finally become the common progenitors of adult LC and PTM 79. Regarding EC, although some studies proved the early appearance of EC in prenatal male gonad 73, 84, so far there have been few studies that focused on EC development. Like the previous murine studies 85, 86, scRNA-seq data on human the fetal testis also revealed two distinct types of macrophages in the fetal testis, of which one is named *SIGLEC15^+^* fetal testicular macrophages (osteoclast-like) and the other is *TREM2^+^* fetal testicular macrophages (microglia-like) 74. Further study showed that *SIGLEC15*^+^ macrophages were usually located in the interstitium and could interact with EC/mesenchymal cells and play roles in mesonephric endothelial cell migration, while *TREM2*^+^ macrophages (located in the testis cord) interacted with fetal SC and FGC and worked as an immune regulator 74. Unfortunately, the transition from fetal testicular macrophages to postnatal testicular macrophages remained unexplored. Further scRNA-seq-based studies might have the opportunity to reveal the relationship between fetal and infant testicular macrophages.

#### After-birth testicular maturation from underage to adult

##### 1. Overview

Among all scRNA-seq studies on testicular samples, several studies did original scRNA-seq on underage testicular samples 31, 55, 67, 72, 79, 87. And in some other studies 78, 88, previously published underage testicular scRNA-seq data were re-analyzed. Those studies pointed out that huge heterogeneity existed in both spermatogenic cells and somatic cells during the maturation of testes after birth.

##### 2. Spermatogenic maturation

For analyzing the after-birth development of spermatogenic cells, pseudotime trajectory analysis was widely used. In fact, pseudotime trajectory analysis on spermatogenic cells could be divided into three distinct types. First, if we only focused on the normal testis of an adult man, spermatogenesis itself, starting from SSCs and ending at mature spermatids, was a continuous process running along a time axis of maturation 89. Hence, the complete spermatogenesis process could be analyzed using pseudotime trajectory in scRNA-seq data. In 2018, Guo et al. did this task and showed there was only one no-branch pseudotime trajectory in spermatogenesis of healthy male 31, which started with SSCs, passed diff_SPGs, early and late SPCs, then round and elongated spermatids and ended up with testicular sperms (also known as mature spermatids 90). The second type of pseudotime trajectory analysis on spermatogenic cells is to extract and re-cluster a certain type of germ cells and do pseudotime trajectory analysis on this type of germ cells. For instance, Sohni et al. did pseudotime trajectory analysis on SPGs and found a minor group of SPGs called “transitional cells” which was the linkage between SSCs and diff_SPGs during SPGs differentiation 67. Besides, Guo et al. did pseudotime analysis on SPGs and reported a five-stage development of SPG, starting from state 0 (markered by *PIWIL4*, similar to the above-mentioned state f0 in the fetal testis), then state 1 (*ID4*, *GFRA1* expressed), followed by state 2-3 (marked by* KIT* and *MKI67*) and finally reaching state 4 (marked by *STRA8*) 31, 42. By comparing these 5 stages with the results from Wang et al. 76 and Sohni et al 67., we could conclude that state 0 and 1 were two types of SSC (0 was more naïve and fetal-like, and resembled the “SSC-1” in Sohni's work, while 1 resembled “SSC-2” in Sohni's work), and state 2-3 were diff_SPG while state 4 were diffed_SPG. Third, pseudotime analysis could also be used to study the maturation of germ cells from underage to adult. In fact, the discovery of state 0 was based on pseudotime analysis on the combination of infant and adult spermatogenic cells, from which they found this special state of SPGs (which was close to the infant germ cell cluster) in adult 31. Sohni et al. further re-clustered infant germ cell cluster into three subclusters, including primordial germ cells (PGCs)-like cells (PGCLs) (marked by *POU5F1* and *NANOG*), Pre-SPG1 (marked by *DOCK8* and *SERINC2*) and Pre-SPG2 (marked by *COL1A2* and *TIMP2*) 67, 91. And further pseutotime trajectory showed that the developmental order of fetal/infant/adult naïve germ cells was PGCL→Pre-SPG1→Pre-SPG2→adult SSC subcluster (SSC-1) 67. During puberty, the spermatogenic status changed obviously with age, and pseutotime trajectory analysis of a series of testicular scRNA-seq data from 1- to 25-years old males showed the dynamic changes of germ cells at different ages 72. In detail, during the infant and child period (1 and 7 years old), there were only SSC in testes (also known as undifferentiated SPG or SPG state 0 and 1, which all referred to the naïve status of SPG) 72, and this finding was further validated by scRNA-seq data on testes from 2- and 5- years old males 55. At year 11, both diff_SPG and SPC appeared, while spermatid appeared at 13 years old samples 72. Zhao et al. further showed that diff_SPG had already existed in the 8-year-old testis but SPC was still absent at that time 55. The first maturity of spermatogenesis was observed at 14 years old, by which time the proportion of each type of spermatogenic cells was almost the same as adult testes 72. In terms of molecular signaling, Activin pathway was downregulated in SSC but was obviously upregulated during spermatogonia differentiation 72. In a word, scRNA-seq together with pseutotime trajectory analysis is very powerful in analyzing the after-birth maturation of spermatogenic cells, especially in the identification of new cell subclusters and finding new stages of cells.

##### 3. Somatic maturation

For somatic cells, the development of Sertoli cells has been fully analyzed by scRNA-seq analysis. By integratedly analyzing testicular scRNA-seq data of five young adult with normal spermatogenesis (age from 23 to 31 years), three children (age 2, 5 and 8 years) as well as two adolescents (age 11 and 17 years), Zhao et al. revealed that the normal maturation of testicular Sertoli cells consisted of three stages (termed as stage_a, b and c) 55. In detail, stage_a (marked by *JUM*) was the majority of Sertoli cells at infant time (including the neonatal testis) and gradually decreased with the age increasing. While stage_c (marked by *DEFB119*) appeared at the time of puberty (after 11 years old) and became the dominant type of Sertoli cells in adult people. Besides, they also found that the Wnt signaling pathway should be the key of regulating Sertoli cell maturation 55. Such findings are outstanding because it focused on the normal development of postnatal somatic cells and revealed not only the phenotype development of Sertoli cells but their related molecular mechanisms as well. Similarly, Guo et al. also found two immature stages of SC, named immature 1# (marked by *PDPN*) and 2# (with higher expression of *TOMM7* and* ATP5E*), which finally merged into mature SC after 11 years old 72. By comparing the differentially expressed genes (DEGs) of stage_a/b SC (from Zhao's study) versus those of immature 1#/2# SC (from Guo's study), we concluded that stage_a was similar to immature 1# while stage_b was closer to immature 2#. A huge difference between these two studies is that Guo's study showed 1# and 2# as two independent paths of immature SC, which will merge into mature SC after 11 years old. Zhao's study, however, showed a potential sequence of stage_a, b and c, (from a to be to c) by both analyzing the proportion of these three cells at different ages and analyzing the functions of these three cells. In detail, they found that before the appearance of mature SC, the ratio of SC a to SC b decreased with age, suggesting there might be a transition from a to b 55. But was this change of ratio caused by “transition from a to b” or by “proliferation of b/death of a”? Enrichment analysis and cycle-specific gene analysis further showed that stage a SC were the most proliferative and had more features as progenitors, which supported the hypothesis that stage a, b and c were three consecutive development stage of SC 55. Since Zhao's study was more comprehensive in terms of SC, hereinafter we chose the nomenclature of SC from Zhao's study 55.

Moreover, Mahyari et al. found that in adult human testes, the maturation of Leydig cells also consisted of three types including progenitor LCs (PLCs), immature LCs (ILCs) and mature LCs (MLCs) 78. And pseudotime analysis confirmed that the maturation of LCs started from PLCs, followed by ILCs and ended at MLCs 78. However, in underage testes, the LCs seemed to be a unique type which was totally separated from the above-mentioned three adult LC types 78. In line with that finding, using pseudotime analysis on both infant and adult LCs, another study also found that infant LCs and adult LCs appeared at two different ends of the trajectory, indicating that the progress of LCs from underage to adult might be a discontinuous alteration 67. Further, Guo et al. for the first time showed that the so-called “infant or child LCs” might actually be the common progenitors of LC and PTM 72, which will finally develop into both LC and PTM during puberty 72. As for PTMs, although the developmental trend was similar to LCs (because they were homologous), pseudotime analysis of scRNA-seq data identified a special subtype of adult PTMs which was clustered together with neonatal PTMs and showed a neonatal-like gene expression characteristic 67. For the molecular signaling involved in the maturation of LC and PTM, Guo et al. found that the common progenitors expressed high level of genes associated to “transcription” signaling, and the enriched pathways went to cytoskeleton/ cell adhesion signaling (if the progenitor differentiated into PTM) or secretion signaling (when it differentiated into LC) 72. So far, the development and maturation of other types of somatic cells, especially testicular ECs and immune cells, have not been fully revealed. Future scRNA-seq studies should pay special attention to the differences of these rare somatic cells between underage and adult human testes.

Based on the published studies, we can conclude that during the maturation of after-birth testes, huge differences existed in both somatic cells and germ cells between underage and adult testis in terms of cell subtypes, expressed markers and regulating mechanisms.

#### Testicular senescence from adult to aged males

##### 1. Overview

Unlike the deep studies on differences between underage and adult spermatogenesis, few scRNA-seq researches focused on the changes in aging testes. Until last May, Nie et al. published the world's first testicular scRNA-seq study based on the comparison between young and old adult men 75. In this study, by integrated analysis of 4 young adults (age 17-22 years) and 8 older adults (age 62-76 years), this team found huge age-related changes in both spermatogenic cells and somatic cells.

##### 2. Germ cell senescence

In terms of germ cells, they found that spermatogenic cells were affected by age in an inconsistent way (some old people still have complete spermatogenesis, while others showed impaired spermatogenesis), and in old males with obviously impaired spermatogenesis, the decreased number of SSC might be one of the causes of age-related spermatogenic dysfunction 75. Mechanistically, using Cellchat analysis, a powerful function of scRNA-seq data to detect cell-cell communications 92, Nie et al found that Activin and KIT signaling pathways weakened between SCs and SSC/diff_SPGs, which might lead to damaging effects on germ cells 75.

##### 3. Somatic cell senescence

For somatic cells, scRNA-seq analyses revealed pan-somatic cell changes, including inflammation changes of SCs, loss and testosterone decline of LCs, decreased contraction and integrity of PTMs-based tubular walls and the senility of both ECs and Macrophages 75. Mechanistically, Nie found that for SCs, metabolic signaling was significantly altered in aged males, while in senescent LCs, key components of the Hedgehog pathway were decreased 75.

##### 4. Young infertile testes showing onsets of senescent process

For some infertile males who were still young, Alfano et al. found that their testes showed early onsets of **s**enescent process 88. Such abnormal senescence was observed in almost all somatic cells of idiopathic germ cell aplasia, and was characterized by the upregulation of proteins of innate immunity, the overexpression of* UBA52*/*NACA* and decreased pathways of amino acids metabolism 88. This phenomenon suggested that the **s**enescence of human testes was not only associated to the exact age of male, but affected by their pathological status as well.

#### Transitions of SC/LC/PTM in the context of the whole life cycle

**Figure 3:**
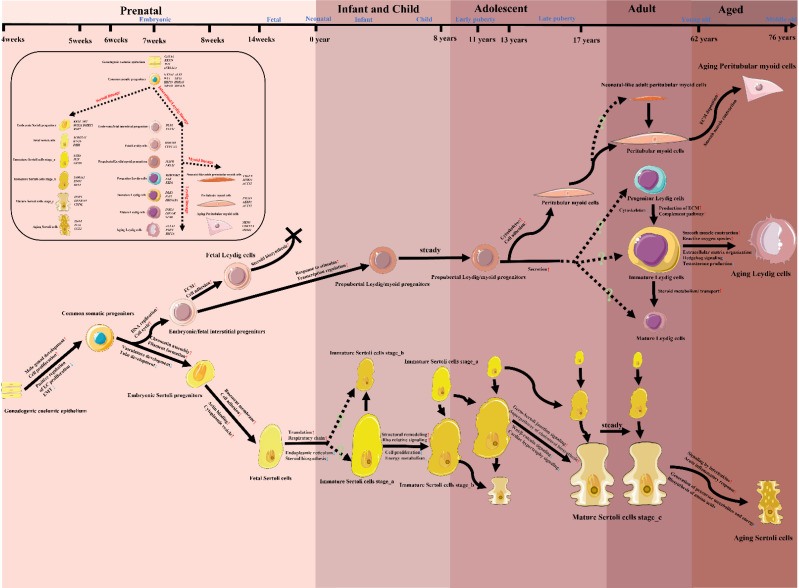
A model map of the potential developing paths of Sertoli cells/Leydig cells/Peritubular myoid cells in the whole life cycle, together with changing cell functions/pathways and highly expressed markers. The information of this diagram was retrieved from studies listed in Table 1, especially from 55, 67, 72-75, 78-80. Icons of various cells in this figure were downloaded from Servier Medical Art (https://smart.servier.com/) or redrawn based on the elements of Servier Medical Art. EMT, epithelial-mesenchymal transition; ECM, extracellular matrix.

So far, there has been limited scRNA-seq study that focused on the all-age process of the origin, development, maturation and senescence of a certain type of testicular cells by analyzing prenatal, infant, children, adolescent, adult and aged testicular samples. Due to the profound studies on the transitions of SC/LC/PTM and the low inconsistency among these studies, we tried to summarize the development and changes of these three main somatic cells in the context of the whole life cycle (from embryotic to aged) in **Figure 3**, based on the current evidence retrieved from human testicular scRNA-seq studies 55, 67, 72-75, 78-80. Future studies should try to integrate the transcriptional phenotypes of testicular cells from all periods of the life cycle, and do a coherent study on the other types of testicular cells, especially testicular immune cells, to which less attention was paid.

### ScRNA-seq mapped the testicular microenvironment in infertile males but with complete spermatogenesis

#### Obstructive azoospermia

Obstructive azoospermia was clinically diagnosed by semen tests (no sperm in ejaculates) and the existence of excurrent duct obstruction 93. According to traditional opinion, OA patients were with rather conserved spermatogenesis 94. Therefore, in traditional studies, due to the difficulty in obtaining completely healthy and fertile male testicular samples 95, 96, researchers would like to choose OA samples with confirmed normal histopathologic features as the controls 97-100. In testicular scRNA-seq studies, this workaround was also widely used 55, 66, 76. Although we could conclude that these OA control samples were with complete or full spermatogenesis because of the histopathologic confirmation, could these OA testes strictly be called as “normal” testes? In the past, due to the limitation of research methods, this question was not fully answered. By the combination of healthy and OA testicular scRNA-seq data, Chen et al. revealed an obvious loss of late spermatids and an increase of early SPCs in OA testes compared with testes from the healthy man 56. Mechanistically, the changes were found in both germ cells and somatic cells. First, SPCs of OA testes might suffer from the weakening meiotic process and enhanced apoptosis, which eventually leading to the loss of spermatids 56. As for somatic cells, PTMs might be the matchmaker between obstruction-caused pressure and the damage of spermatogenesis 56. This study partially solved the unsettled issue and suggested that although the spermatogenesis of OA testes was functionally and pathologically normal, it was not technically normal. Meanwhile, this study only used scRNA-seq data of post-epididymitis and post-vasectomy OA testes. Is there a difference in testicular function among OA patients with various causes (e.g., congenital bilateral absence of the vas deferens and post-vasectomy)? Whether the changes of OA testes found by scRNA-seq are obstructive time-dependent? These questions remain to be answered.

#### Ejaculation dysfunction

Ejaculation dysfunction contains a wide range of situations in which the normal ejaculation was disordered or even failed 101. Infertility caused by ejaculatory dysfunction or retrograde ejaculation could also be accompanied by testes with complete/full spermatogenesis. Using testicular scRNA-seq data of a patient with retrograde ejaculation (post-colectomy), Mahyari et al. pointed out that the characteristics of cell score residuals (a statistic reflecting testicular cells' pattern) of the retrograde ejaculation sample and adult control samples were alike, which indicating that the patient with retrograde ejaculation had undisturbed spermatogenic status and could be considered as another type of control samples 78. Nevertheless, the above-mentioned scRNA-seq study did not especially focus on ejaculation dysfunction, so the evaluation of testicular changes of ejaculation dysfunction was not that comprehensive. Further scRNA-seq studies should take several situations of ejaculation dysfunction, including “total anejaculation”, “situational anejaculation” 102 and “retrograde ejaculation”, into consideration, in order to profoundly reveal the potential alterations caused by ejaculation dysfunction.

### ScRNA-seq revealed cellular and molecular disorders in patients with spermatogenic dysfunctions

#### Non-obstructive azoospermia

Spermatogenic dysfunction was characterized by the impairment of intratesticular spermatogenesis, and NOA is the most severe condition of it 103. NOA was clinically diagnosed with no sperm in ejaculates along with evidence reflecting/causing the impairment of testicular spermatogenesis (e.g., elevated FSH or history of chemotherapy) 104. Maybe due to the relatively easy availability of NOA testicular samples (a large number of NOA patients need testicular sperm retrieval surgeries 105, 106), so far there have been seven studies that did their own scRNA-seq on at least one NOA testicular sample (**Table 3**). It should be noted that in this part (**Table 3**) we only discussed NOA samples without evidence of chromosome abnormality (NOA patients with chromosome abnormality will be discussed separately). Among these studies, over 70% (five) were conducted by Chinese research teams. Interestingly, due to the heterogeneity of NOA, the pathological status of scRNA-seq NOA samples used in each study varied across studies. Besides, maybe due to the decrease or lack of germ cells in the NOA testis, most of these studies focused on changes in somatic cells, especially SCs and LCs. Notably, most of the scRNA-seq studies did analyses on NOA samples in the following two ways: 1) identifying DEGs (in a certain type of cells) between control and NOA testes, and 2) finding new/major subtypes of a certain cell in NOA. For instance, Zhao et al. found that in iNOA patients, Sertoli cells were basically in stage a and stage b (mentioned above), and they revealed iNOA SCs showed different gene expressions pattern compared with normal testes 55, which were independently confirmed by Wang et al. 76 and Chen et al. 56. In terms of somatic cells, two main somatic cells of the human testis, SCs and LCs, showed similar status in NOA patients, both were immature but with enhanced proliferative/divisive ability 55, 78, which were in line with the fact that immature somatic cells have proliferative ability 107, 108. Alfano et al. further found that in SCOS (the severe pathology of NOA), the transcriptional and phenotypic features of LC were more like pre-pubertal LC, indicating the repressed maturation of LC in NOA 88. As for germ cells in the NOA sample (if it had germ cells), Cst3 mediated autophagy might play an important role in the development of NOA by harming SSC maintenance (the ability of SSC to keep its population by self-renewal 109), and knockdown of this gene might cause the impairment of SSC which might further lead to spermatogenic dysfunctions 110.

Unfortunately, maybe due to the reduction or absence of germ cells in the NOA testis, most of these studies merely focused on somatic cells, which made the changes of residual spermatogenic cells in NOA patients largely unknown. Future studies could especially select samples with spermatogenic arrest or hypospermatogenesis to do scRNA-seq, which will give a chance to solve this problem.

#### Cryptozoospermia

In the area of Oligo-astheno-teratozoospermia (OAT), so far there have been few scRNA-seq data reported. This phenomenon is easy to understand because the scRNA-seq data was based on testicular tissue which is usually dissected in sperm retrieval surgeries such as TESE or microdissection TESE (mTESE) 111, and for OAT patients, many of them could use ejaculated sperm to do *in vitro* fertilization (IVF). Only severe OAT patients might need to undergo testicular sperm retrieval surgeries 112. Cryptozoospermia, which was characterized by the appearance of isolated sperms in semen under the microscopic examination after centrifuging 106, could be considered as the transitional situation between severe oligozoospermia and azoospermia. The previous study pointed out that for cryptozoospermia patients, surgically retrieved testicular sperm was better than ejaculated sperm when doing intracytoplasmic sperm injection (ICSI) 113, which could also provide more convenience for obtaining testicular samples to do scRNA-seq. Using three cryptozoospermia testicular samples versus three controls (OA samples), Persio et al. profoundly revealed the extensive changes of testicular microenvironment in cryptozoospermia patients 66. Deeper changes occurred in spermatogenic cells, including the reduction of germ cells such as pachytene spermatocytes and beyond, the changes of transcriptional profiles of all-type spermatogenic cells, an increase of PIWIL4 positive spermatogonia (representing the most naïve germ cells 114, just like the above-mentioned state 0 SPG or SSC-1), and a decrease of A_dark_ SPGs (reserved SSCs 115) and UTF1 positive SPGs (a SSC/undifferentiated SPG marker 116) 66. More importantly, an enhanced and prolonged expression of *EGR4* was noticed in SPGs (also resembled the SSC-1 cells in Sohni's study 67), which could act as a transcriptional factor and inhibit the expression of *UTF1* in part of SPGs from cryptozoospermia patients 66. Besides, scRNA-seq data also revealed perturbed somatic cells in cryptozoospermia, including an obvious increase of PTMs (including fibrotic PTMs) and macrophages, a deep transcriptional change of PTMs, an elevated ratio of MUSTN1 postitive (a pan-musculoskeletal cell marker 117) blood vessels and the gathering of CD3 T cells around blood vessels 66. Besides, two crosstalk networks between SPGs and adjacent microenvironments including “SPGs-*FGFR1*/*3*-*FGF2*-pachytene/diplotene SPCs” and “SPGs-*ACKR2*-*CCL2*/*3*/*4*/*5*/*3L1*/*14*-EC/immune cells/perivascular cells” were detected in cryptozoospermia rather than normal patients 66.

#### Trans-females after gender-affirming hormone therapy

The testis removed from patients receiving sexual reassignment surgery was another important tissue source for scRNA-seq studies 43, 72. Of course, if a physically healthy patient did not receive any medical treatment before transsexual surgery, the removed testis was definitely an ideal sample of the “normal” group. Unfortunately, many transgender individuals received gender-affirming hormone therapy 118, which might profoundly impair the spermatogenesis of their testes 119. Guo et al. drew the single-cell transcriptional profiles of two trans-females who received long-term (over a year) testosterone antagonist and estradiol treatment before transsexual surgeries 72, and found huge disorders in both germ cells and somatic cells. In terms of germ cells, although they still existed in both donors, the proportions of advanced spermatogenic cells including spermatids, SPC and diff_SPG were extremely low or even absent, and such changes stopped at the level of undifferentiated SPG (or SSC, or state 0 & 1 SPG as mentioned above) 72, indicating that testosterone inhibition might have less impact on SSC itself at the transcriptional level but could stop its differentiation. We found that such status was similar to the testis of prepuberty boys, for whom diff_SPG and more developed germ cells did not appear before 8 years old 55. As for somatic cells, SC was found to be immature with higher expression of *AMH* and *HES1* in these two patients, and the transcriptional profiles of SC were more like pubertal males (stage_b SC was predominant during this period 55) rather than adults 72.

### ScRNA-seq detected impairment of testicular somatic cells in patients with chromosome abnormalities

#### Klinefelter syndrome

Klinefelter syndrome (KS) makes up the most common part of male chromosome disorders 120, which is also widely studied by scRNA-seq studies 55, 78, 121. Importantly, usually the studies on KS patients were considered as a part of works on NOA patients because of the popular azoospermic phenotype 122. Nevertheless, due to the special karyotype of KS (one or more extra X 123), the changes and relative mechanisms in KS testes were distinguishable from the above-mentioned iNOA patients 124. Interestingly, for scRNA-seq data of KS testicular samples with the presence of germ cells, there seemed to be no huge changes at the transcriptional level of KS germ cells compared with normal germ cells 121. But such a conclusion was drawn indirectly based on the co-clustering of KS germ cells with normal germ cells, rather than differential expression analysis (because there was only one KS sample sequenced with only 39 germ cells identified in Laurentino's study 121). Unlike the other type of male infertility (in which the scientist could deeply analyze germ cell changes), Laurentino's study was almost the “best” work of KS germ cells because most of the scRNA-seq samples on KS from other teams lacked spermatogenic cells and showed a SCOS or even tubular atrophy pattern 55, 78. Although previous study pointed out that spermatogenic cells were not entirely lost among all KS patients 125, we found it was hard to accurately get one sample with germ cells to do scRNA-seq. According to our experience, for KS patients, if the sample was coming from a random biopsy, we can't guarantee there were germ cells in it. And if the sample was coming from TESE or mTESE surgery (which we could judge the spermatogenic status by the form of tubules during surgeries), although it was possible for us to find a sample that contains germ cells or even spermatozoa, most of such “good” samples should be used for finding sperms and ICSI (since such good samples were quite rare and precious), so it was unlikely to have an extra “good” sample for scientists to do further studies including scRNA-seq. By combining scRNA-seq with bulk RNA-seq data and IHC staining, Winge et al. found that such germ cell loss in KS patients might start as early as in the fetal testis 126. And another study found that *KIF2C* might be a germ-cell-related gene to regulate spermatogenic development in KS testes 127. Frankly, based on the current evidence from scRNA-seq data, it's too early to answer the question that what was the transcriptional change in the residual germ cells of KS patients.

As for KS somatic cells, scRNA-seq data has offered us abundant findings which we didn't know before. SCs were found to be the most affected somatic cells in KS testes 126. In detail, KS SCs were almost stage_b SCs and showed altered energy metabolism characteristics such as enhanced expression level of glycolysis and oxidative phosphorylation-related genes but rather low triglyceride metabolism level 55. Huge transcriptional changes were observed in KS SCs including the upregulation of immune-related genes such as *B2M* 128 and* MIF* 129, the decreasing of sex hormone -regulating gene *GNRH1* and the increasing expression of X-linked genes 55, 78. Notably, Mahyari et al. found a special subpopulation of KS SCs which showed lacking expression of *XIST*, which could partially explain the X-linked genes' enhancement in SCs 78. The involvement of immune activation in KS testes found by Zhao et al. 55 were also validated in peripheral blood of KS patients 130, indicating the profound impact of immune-related pathogenic factors in the pathogenesis of KS. Similarly, the proportion of immature LCs was also increased in KS LC population and more LCs were found to undergo division/development (with higher expression level of RNA processing/splicing-regulating genes such as *CCNL1*, *DDX17*, *PNISR* and *FUS*) 78. Interestingly, when comparing KS-NOA somatic cells with iNOA somatic cells, both shared and heterogenous features were found. For example, the expression of β-catenin protein level was both significantly enhanced in KS-NOA and iNOA SCs, indicating a shared pattern of activated Wnt/β-catenin pathways in both type of NOA compared with normal adults 55. While *SERPINE1*, a tissue plasminogen blocker, was highly expressed in KS immature LCs but not in iNOA LCs, indicating the heterogenous pathogenesis of KS-NOA compared to NOA with other etiology 78.

#### Y chromosome microdeletion

The testicular evidence got from single-cell studies on patients with Y chromosome microdeletion was limited and less than the evidence from bulk studies 131, 132, especially for AZFc microdeletion (the most common form of Y chromosome microdeletion in male infertility 133). So far, there has been only one scRNA-seq testicular sample with azoospermia factor a (AZFa) deletion reported 55. Unfortunately, this sample was with SCOS pathology, so we can't gather enough data on germ cells of patients with Y chromosome microdeletion. Pseudotime trajectory analysis in the AZFa testis found its SCs were in the early part of stage_c (a form of mature SCs), together with upregulation of *MIF* and *DEFB119* (two important factors in immunoregulation), suggesting the involvement of immune regulating pathways in the etiology of AZFa SCs 55. Considering the limited research on this field, we suggest future scRNA-seq studies should focus on the pathogenesis of azoospermia factor b (AZFb) and azoospermia factor c (AZFc) testes (especially those AZFc testes with germ cells), so as to profoundly reveal the special changes in patients with Y chromosome microdeletions.

### Re-analysis of published scRNA-seq data on human testicular samples met diversified scientific requests

#### Overview

With more and more scRNA-seq data on human testicular samples published, so far there have been plenty of studies that chose to re-analyze those published data in various aspects. **Table 4** summarized the studies that re-analyzed the previously reported scRNA-seq data on human testicular samples. A special case is Chen et al.'s spatial transcriptomic work on testes 134. In that Slide-seq-based study, the authors only employed published scRNA-seq data as a reference for cell-type assignment, and as an input for pseudotime value assignment, which (strictly speaking) were not the real “re-analyses” of scRNA-seq data itself. Therefore, we chose to exclude that study from Table 4. Among the reviewed 48 studies in **Table 4**, the majority of them combined the human testicular scRNA-seq data with other types of scRNA-seq data or bulk RNA-seq/microarray data in order to get integrated results, and most of them chose to experiments such as IHC/immunofluorescence (IF) staining on testicular sections to validate their findings. Hence, in **Table 4** we also offered the important public data (other than human testicular scRNA-seq data) that the study used and listed the experiments/clinical studies or sequencing works conducted on their own. Although those studies had various designs and different findings, we still found universalities among these studies and categorized them into several categories. The detailed classifications as well as representative studies were discussed hereinafter, and many of the studies were with multi-categories.

#### Detecting the transcriptional pattern of interested genes in human testes

As a transcriptome-level technology, the fundamental usage of scRNA-seq was to detect genes' transcriptional patterns such as the mRNA expression level of a certain gene in different types of cells. A typical study was carried out by Yang et al. 135, in which they first found a new variant of *KASH5* by Whole Genome Sequencing (WGS) in an NOA patient, but did not know the detailed *KASH5* expression pattern in the human testis. So, they chose scRNA-seq data (the data used here were previously reported by the same team) to see the expression of *KASH5* and found it to be enriched in SPCs, especially leptotene to pachytene SPCs, and finally validated it by IF staining. This process of “finding target gene” and then “detecting it in human testicular scRNAs-seq data” (sometimes with a third step as “validating genes using experiments”) was quite common for studies with interested genes 136-141. Meanwhile, the interested genes were not restricted to one gene or several genes, the researchers could detect a gene family (such as Interleukins) or a series of function-related genes (such as inflammatory cytokine receptors) in scRNA-seq data 142, which could give researchers a view on macroscale. Moreover, an updated usage of this process was to not only show the type of cells that a certain gene was expressed in, but also show its transcriptional changes during spermatogenesis 143 or among different pathological groups 127, 144. A good example came from He et al.'s work 127, in which they identified four interested genes (*KIF2C*, *MRPS2*, *RPS15* and *TSFM*), and then based on human scRNA-seq data of both KS testes and normal testes, they showed that *KIF2C* were downregulated while *RPS15* were upregulated in KS testes. Additionally, pseudotime trajectories showed a growing tendency of *KIF2C* and a downtrend of *RPS15* with the maturation of spermatogenic cells 127. We need to point out that no matter what method the study used and no matter how complicated the study procedure was, the nature and essence of this type of re-analyzing were to show the expression of given genes in human testes, no matter how these interested genes were found out (by other technologies/experiments, by bulk RNA-seq/microarray data, by scRNA-seq data of other organs/species, by literature reviews or even by the authors' own idea).

#### Indirectly revealing the vulnerability of testes to SARS-CoV-2 infection

Due to the Corona Virus Disease 2019 (COVID-19) pandemic and the high proportion of SARS-CoV-2-related articles in **Table 4** (10 out of 48), we especially discussed the usage of human testicular scRNA-seq data here. As for the usage of testicular scRNA-seq data, most of these studies were actually in the form of “detecting the transcriptional pattern of interested genes in human testes” 145-154, since several important proteins, such as ACE2 155, TMPRSS2 156, Furin 157 and CD147 158, were deeply involved in the entry of SARS-CoV-2. So far, the opinions on this topic have been controversial. In some studies, *ACE2* was reported to be expressed in both germ cells (especially SSCs&SPGs) and somatic cells, including SCs, LCs as well as myoid cells 145, 147, 149, 154. And one study showed a higher positive rate of *ACE2* in testes from infertile patients (OA and NOA) 148. Those results indicated the potential vulnerability of human testes to SARS-CoV-2 infection, especially in infertile men. And some studies even called the human testis a “high-risk organ” for SARS-CoV-2 infection 152. But based on the scRNA-seq-derived evidence that there was no co-expression of *ACE2* and* TMPRSS2* in the human testis, other two studies gave the opposite view that human testes might not be able to be long-term affected by SARS-CoV-2 infection 146, 151. From our point of view, those ten scRNA-seq-based studies only gave indirect evidence based on previously published data. By combing original and published scRNA-seq data on human testes, Liu et al. partially overcame the shortcomings of those studies and for the first time showed an expression of *ACE2* in primordial germ cells of prenatal testes and a downward trend of *ACE2* with the age increasing 159. More importantly, although *ACE2* had the highest expression in SCs, it significantly shrank in SCs of NOA patients 159. But the results of Liu's study were still based on scRNA-seq of patients without histories of COVID-19, which were also indirect. So far, there has been no report of scRNA-seq data on human testes from COVID-19 recovered patients, maybe due to the huge difficulty of sample obtaining. But with the pandemic continuing, there must be patients who simultaneously suffered from COVID-19 and male infertility and needed testicular biopsy or TESE surgery. Therefore, in the future, researchers can use scRNA-seq to compare testicular samples from COVID-19-recovered patients with those from non-COVID-19 patients, which will offer direct evidence of the impact of SARS-CoV-2 on human testes.

#### Finding novel genes involved in normal testicular function and male infertility

Testicular scRNA-seq data could not only be used to draw expression patterns of certain genes in the testis, but could directly be used as the sources of such novel genes. The difference between this type of usage and the type “Detecting the transcriptional pattern of interested genes in human testes” is that this type of study directly used testicular scRNA-seq data as a tool to find novel genes, while the other one only used scRNA-seq data as a tool of validation or display. For example, by comparing the DEGs in SPG between OA and NOA scRNA-seq data, Yang et al. reported a novel SPG-specific gene *ELAVL2* and found it to be significantly downregulated in SPGs of NOA patients 160. Further experiments proved this gene as an important factor in regulating SSC maintenance and apoptosis 160. Like that study, scientists could use testicular scRNA-seq data as a tool to reveal functional genes that may be related to the development of testicular diseases 161-164. We recommend that this type of usage should be “cell-oriented”, which means the researchers should focus on a certain type of testicular cells and try to dig up novel genes or mechanisms of this type of cells, rather than just comparing the differences between disease and control groups without considering cell heterogeneity (in other words don't use scRNA-seq data to do tasks that bulk RNA-seq can do).

#### Comparing conserved and species- or sex-specific transcriptional features

Another important type of re-analyses of human testicular scRNA-seq data is to do species or sex comparisons. For species comparison, so far, the spermatogenesis of mouse 165, mouse lemur 165, chicken 166, cynomolgus macaque 167, sheep 168 and the somatic development of pigs 169 has been fully compared with that of human. The similarities of these studies were that they reported both conserved (especially the conservatism of X chromosome inactivation) and species-specific features among different animals and human. A good example would be Tian et al.'s work, in which they compared the spermatogenesis of four different species (human, mouse, sheep and cynomolgus macaque) 168. In that study, they found the spermatogenesis between sheep and human were partially conserved at the transcriptional level (e.g., similar regulators were expressed in the same stages of spermatogenesis in human and sheep) and further showed the high conservatism of X chromosome inactivation during meiosis among four species 168, which was in line with Lau et al.'s report 167. On the other hand, Rengaraj et al. reported a large proportion of species-specific genes between human and chickens (24 out of 56 orthologs) during the development of male fetal germ cells 166. Additionally, the species comparison (usage type 4) could be combined with detecting certain genes' expression patterns (usage type 1). For example, *INPP4B* was found to be highly expressed in postmeiotic spermatogenic cells in the testis of both human and mice 170, which remained evolutionary conservatism, while *SFTPC* was expressed in late spermatogenesis of human but were absent in murine spermatogenesis 171, which indicated evolutionary divergence. And the tasks of those two studies were using testicular scRNA-seq data to show the expression of certain genes, but such tasks were done cross-species by combining two types of usage. In this area, the spermatogenesis among mammalian species or some other common animals (such as chicken) has been largely compared, but two subjects remain to be discovered. First, are there any highly conserved genes in spermatogenesis between humans and rare species or other non-mammalian animals (which are evolutionarily far)? Second, what's the conserved mechanism of testicular somatic cells' development among species? We believe scRNA-seq analysis will give the answers in the future. Besides, it's well known that ovary is the female counterpart of the testis, thus scientists tried to compare gonads of two genders. The conservatism of germ cells for both genders was observed during meiotic prophase I, such as the high expression of the same marker genes at each stage of primary SPC/oocytes (*TEX19* in leptotene; *SPO11* in zygotene; *BRDT* in pachytene and *H1FOO* in diplotene) 172. Wang et al. even identified shared subtypes of germ cells (SPARC^+^ fetal germ cells) in prenatal testes and ovaries 82. But huge heterogeneity of germ cells was also reported in the expression of X-linked genes, including the high expression of *SMS* in diplotene oocytes but not SPC and the lack of *XIST* in adult testes 73, 172. Both homogeneity and diversity between genders were also reported in somatic cells during scRNA-seq analyses. For example, shared transcriptional expressions of *NR5A1*/*SOX9* in both fetal SC and granulosa cells 73 and shared type of somatic cells (KRT19^+^cells) 82 reflected the homogeneity between genders, while the divergent somatic expression pattern of *DMRT1* (high in SC but not granulosa cells) suggested the diversity 73.

#### Bioinformatic usages including algorithm developing and database/portal establishing

Since developmental biology is one of the most important areas in which scRNA-seq analysis is applied 173, so far there has been a huge accumulation of related raw data deposited online. Therefore, the raw data could then be used to develop or validate scRNA data-related bioinformatic tools, such as bioinformatic algorithms, packages, softwares and databases. Pont et al. developed a method for single-cell based scoring system and visualization of gene set signature, and a scRNA-seq set of normal human spermatogenesis was employed to show the function of this method 174. Recently, Stow et al. developed another scRNA-seq-based bioinformatic tool called SCIFER to analyze long interspersed element-1 (L1) mRNA level from individual L1 loci in single cells, and further employed human testicular scRNA-seq data to show its function 175. Theoretically, the role of scRNA-seq data could either be a developer (to build up the tool) or a validator (to confirm the tool works well or to show how to use it), while the current studies seemed to prefer to use testicular scRNA-seq data as a validator. Moreover, for many life scientists (especially clinicians) who would like to use scRNA-seq analysis to validate their findings or interested genes in human testes, it's too hard for them to start from the raw data due to the high demand for coding skills 176. Therefore, it's more convenient for researchers to use some interact-friendly or “zero-code” online websites/portals. So far there have been several popular interactive online portals containing human testicular scRNA-seq data (listed in **Table 5**), some of which were testis-specific 31, 72, 78, 177, two were human gonad-based (with female's samples and *in vitro* differentiated gonadal cells) in terms of scRNA-seq data 79, 178-180, while the rest were based on various organs with testicular scRNA-seq data included. The function of these online portals was basically Uniform Manifold Approximation and Projection (UMAP)/t-distributed stochastic neighbor embedding (t-SNE) visualization of cells and illustration of the expression patterns of certain genes. Some advanced functions such as pseudotime trajectory of testicular cells or enrichment analysis are only supported by few portal 78. As a result, bioinformatic engineers should pay more attention to the realization of integrated analysis and comprehensive analysis through interact-friendly (non-code) website tools.

## Conclusions and future perspectives

The main purpose of the current review is to gather the updated evidence on human testicular samples revealed by scRNA-seq data, in order to gain a “single-cell” view of both normal and pathological development of the testis. So far, there have been a large number of novel findings in terms of normal spermatogenesis, age-related testicular microenvironment's development and changes, testis-related male infertility, as well as new bioinformatic methods suitable for testicular scRNA-seq data analysis. In terms of normal status, the whole development process of spermatogenic cells (from the most naïve primordial germ cells to testicular spermatozoa), together with their transcriptional patterns at each stage were largely revealed (including their similarity and differences among species). The researches on normal somatic cells were basically restricted to SCs, LCs and PTMs, while fewer scRNA-seq-based findings about human testicular macrophages, ECs or other rare cells (e.g., T cells) were reported. Additionally, numerous transcriptional changes and potential mechanisms have been revealed in disease data including spermatogenic dysfunction, OA, ejaculatory disorder and chromosomal abnormalities, giving us insight into the heterogenous pathogenesis of male infertility and providing us with innovative targets of treatments. Last but not least, more and more publicly available testicular scRNA-seq datasets give researchers more opportunities to do their individualized data analysis for different research needs. We believe this review will not only pave the way to the transcriptionally understanding of both normal and abnormal spermatogenesis, but also be a reference for the future usage of scRNA-seq analysis in the human testis.

One of the most prospective research highlights in this area is the combination of testicular scRNA-seq analysis with spatial transcriptomic analysis of the testis. Due to the dissociation of testicular cells in scRNA-seq, it could neither spatially illustrate spermatogenic cells in seminiferous tubules, nor analyze the spatial interaction between somatic and spermatogenic cells 134. To solve this problem, Chen et al employed the novel technology “spatial transcriptomic analysis” to create spatial atlas that spatially illustrate testicular gene expression at near-single-cell resolution in the human testis 134. More importantly, Garcia-Alonso et al did integrated analyses of the development of male gonads by combining scRNA-seq and spatial transcriptomics 74, which was more comprehensive and in-depth. We believe that the integration of these two approaches will improve future researches of the human testis and male infertility, allowing for a more complete and nuanced understanding of the functional/dysregulated spermatogenesis in the human testis. So far, there has been a limited number of such integrated studies, leading to a novel “Blue Ocean” of testicular researches.

Although scRNA-seq analysis has proved the existence of immune cells, such as macrophages (both M1 and M2), T cells, mast cells, or even B cells, in testicular microenvironment, the role of these minor cells and their transcriptional patterns in the development of male infertility remain unknown.

Meanwhile, more types of testicular diseases, including cryptorchidism, AZFc deletions, azoospermia after chemo/radiotherapy, as well as teratospermia, should be focused on and will benefit from the further usage of scRNA-seq technologies. Due to the high cost of scRNA-seq, few of the above-reviewed articles were directly out of clinical purpose. With the development and cost reduction of this technology, clinical usages such as diagnosis, classification, prediction of sperm retrieval or the effect of hormone treatment might come true.

## Figures and Tables

**Table 1:** Studies containing originally reported scRNA-seq data of human testicular biological samples

Author	Country^*^	Year	Techniques	Conditions	No. of donors	No. of cells	References
Guo et al.	China	2015	Tang method	Prenatal	9	197	30
Guo et al.	USA	2017	Fluidigm C1	Normal	5	175(92 filtered)	42
Guo et al.	China	2017	Tang method	Prenatal	5	14	181
Neuhaus et al.	Germany	2017	Tang method/Shallow RNA-seq	Normal spermatogenesis	1	2	43
Li et al.	China	2017	Modified smart-seq2	Prenatal	12	1204(1068 filtered)	73
Guo et al.	USA	2018	10× Genomics	Normal (one from 17-year-old male)/Infant	5	7830 filtered	31
Vértesy et al.	Netherlands	2018	Smart-seq2	Prenatal	1	9	182
Wang et al.	China	2018	Modified Smart-seq2	Normal/OB/NOA	10	3243(3028 filtered)	76
Hermann et al.	USA	2018	10× Genomics/Fluidigm C1	OB/Normal	16	635 for C1, 36451 for 10x (including 1 experiment with a 1:1 mixture of human and mouse cells)	68
Sohni et al.	USA	2019	10× Genomics	OB/Neonatal	4	33585 filtered	67
Laurentino et al.	Germany	2019	10× Genomics	KS	1	3289 filtered	121
Xia et al.	USA	2020	inDROP	OB	2	2554 filtered	70
Zhao et al.	China	2020	10× Genomics/ BD Rhapsody	OB/iNOA/KS/Juvenile/AZFa deletion	17	88723	55
Liu et al.	China	2020	Modified STRT-seq	NOA	2	61 Sertoli cells	159
Han et al.	China	2020	Microwell-seq	Prenatal	2	13211 filtered	183
Guo et al.	USA	2020	10× Genomics	Juvenile/trans-females	6	19223(12854 filtered)	72
Shami et al.	USA	2020	Drop-seq	Normal	4	13837 filtered	184
Tan et al.	USA	2020	10X Genomics	OB	2	11159(8916 filtered)	69
Chitiashvili et al.	USA	2020	10× Genomics	Prenatal	5	24929	178
Hwang et al.	USA	2020	10× Genomics	Prenatal	3	16429 filtered	185
Persio et al.	Germany	2021	10× Genomics	OB/Cryptozoospermia	6	28690 filtered	66
Wang et al.	China	2021	Modified Smart-seq2/STRT-seq	NOA	1	480(432 filtered)	110
Mahyari et al.	USA	2021	10× Genomics	iNOA/Ejaculatory dysfunction/KS	4	10856	78
Bhutani et al	USA	2021	Smart-seq2	Normal	2	466	186
Alfano et al.	Italy	2021	10× Genomics	OB/iNOA	4	5200 filtered	88
Guo et al.	USA	2021	10× Genomics	Prenatal/Infant	2	6992 filtered (W12 and M5)	79
Chen et al.	China	2022	Slingleron GEXSCOPE^TM^	OB/iNOA	2	3844	56
Nie at al.	USA	2022	10× Genomics	Normal (young adult)/Aging	12	44657 filtered	75
Cheng et al.	USA	2022	10× Genomics	Prenatal	6	89477(72257 filtered) including 4 fetal adrenal glands and 1 4wpf urogenital ridge	80
Wang et al.	China	2022	10× Genomics/modified STRT-seq	Prenatal	11	31006 for 10x 709 for modified STRT-seq	82
Voigt et al.	Canada	2022	10× Genomics	Infant/Juvenile	3	29125	87
Wang et al.	China	2022	10× Genomics	Prenatal	4	53508 filtered for 4 male and 4 female	84
Garcia-Alonso et al.	UK	2022	10× Genomics	Prenatal	22	13381	74

*According to the first authors' information Note: OB, obstruction; (i)NOA, (idiopathic) non-obstructive azoospermia; KS, Klinefelter Syndrome; AZFa, Azoospermia factor a; wpf, weeks postfertilization; W12, 12 weeks post-fertilization; M5, 5 months post birth. Note2: Normal referred to normal adults; Prenatal referred to the fetus and/or embryo; Neonatal referred to several days after birth; Infant referred to several months (upto 1 year and 13 months) after birth; Juvenile referred to all underages (under the age of adult), and Aging referred to >60 years old. Note3: The information of scRNA-seq data/samples in this table referred to the information of the original and first-time reported cases in each study. For example, Guo et al. integrated seven cases in 79, but five of them were first reported by Chitiashvili et al. 178 (these two studies were from the same research team). So, in the item of Guo's study, we only summarized the information of the rest two cases (w12 and M5) which were reported for the first time.

**Table 2:** Representative cell types/subtypes and candidate markers for adult testicular scRNA-seq data

Cell classifications	Cell types	Sub-types	Candidate markers	References
Spermatogenic cells			*DDX4*, *MAGEA4*	66, 75, 76
	SPG			
		SSC	*FGFR3*, *ID4*, *UTF1*, *GFRA1*, *HMGA1*, *PIWIL4*	31, 55, 56, 75, 76
		Diff_SPG	*KIT*, *MKI67*, *DMRT1*	55, 56, 76
		Diffed_SPG	*STRA8*	55, 76
	SPC			
		L.SPC	*DMC1*, *SPO11*, *SYCP3*, *SYCP2*, *SYCP1*, *TEX12*, *RAD51AP2*, *SCML1*	55, 76
		Z.SPC	*SYCP3*, *SYCP2*, *SYCP1*, *TEX12*, *SPO11*, *DMC1*, *RAD51AP2*, *TDRG1*	55, 76
		P.SPC	*OVOL1*, *OVOL2*,* CCNA1*, *CCDC112*	55, 76
		D.SPC	*OVOL1*, *OVOL2*, *CCNA1*, *AURKA*	55, 76
		Meiotic (1/2) and Secondary SPC	*ACR*, *C9orf116*, *SLC26A3*, *SIRPG* might express (Low specificity)	55, 66, 76
	Spermatid			
		Round (early) Spermatid	*TEX29*, *SIRT1*	55, 66, 76
		Elongated (late) Spermatid	*PMR1*,* PMR2*, *PMR3*, *SPEM1*	55, 66, 75, 76
Non-immune somatic cells			*VIM*	31, 75
	Sertoli cells		*SOX9*, *AMH*, *WT1*	55, 67, 73, 76
	Leydig cells		*IGF1*, *DLK1*, *INHBA*, *INSL3*, *HSD17B3*	56, 75, 76, 88
	Endothelial cells		*VWF*, *PECAM1*	31, 55
	Myoid cells		*ACTA2*, *MYH11*	31, 75
		Peritubular myoid cells (PTM)	*ACTA2*, *MYH11*	55, 56, 66, 76
		Perivascular smooth muscle cells	*NOTCH3*, *FAM129A*, *MUSTN1*	55, 66, 75
		Fibrotic PTM	*CLEC3B*, *CFD*	66
Immune cells			*PTPRC*	55
	Macrophages		*CD14*, *CD163*, *CD68*	31, 55, 76
		Macrophage M1	Not reported	78
		Macrophage M2	Not reported	78
	Mast cells		*TPSAB1*, *TPSB2*	55, 56, 164
	T cells		*CD3D*, *CD3E*, *CCL5*	55, 66, 88, 164
		Tissue-resident cytotoxic T cells	*CD8*, *CD69*	88
	B cells		Not reported	78

Note: SPG, spermatogonia; SSC, spermatogonial stem cell; diff_SPG, differentiating SPG; diffed_SPG, differentiated SPG; SPC, spermatocyte; L.SPC, Leptotene SPC; Z.SPC, Zygotene SPC; P.SPC, Pachytene SPC; D.SPC, Diplotene SPC

**Table 3:** Studies with original scRNA-seq data on human testes from NOA patients without evidence of chromosomal abnormality

Year	No. of NOA donors	Age	Technique on NOA samples	Cell numbers of NOA samples	Pathology of NOA samples	NOA etiology	FSH	Testicular volumes	Main findings on NOA spermatogenic cells	Main findings on NOA somatic cells	Reference
2018	1	24	Modified Smart-seq2	174 total	SCOS	Idiopathic*	Unknown	Unknown	Not focused	1.Identifying DEGs of somatic cells (SC, PTM and LC) between NOA and control samples 2.The enrichment of γH2AX signal in NOA somatic cells, indicating their DNA damage response	76
2020	3	26/31/32	BD Rhapsody	20507 total	SCOS	Idiopathic	Increased (based on average data)	Unknown	Not focused	1.Sertoli cells in iNOA patients showed an immature phenotype and suffered from maturation arrest 2.Inhibition of the Wnt signal pathway could reverse the immature status of iNOA Sertoli cells and enhance their assistance to spermatogenic cells	55
2020	2	29/30	Modified STRT-seq	61 Sertoli cells	Spermatogenic Arrest	Unknown	Unknown	Unknown	Not focused	1.SARS-CoV-2-related gene *ACE2* was significantly downregulated in NOA Sertoli cells	159
2021	1	36	Modified Smart-seq2/STRT-Seq	432 total	Hypospermatogenesis*	Idiopathic*	Unknown	Unknown	1.Identifying changes of autophagy-related genes in NOA germ cells, such as the upregulation of *SQSTM1* and the downregulation of *LC3A* in spermatids 2.Cst3-mediated autophagy could help with SSC maintenance by regulating SSC maintenance-related genes (*Oct4*, *Id1* and *Nanos3*) 3.Inhibition of Cst3 disturbed meiosis and spermatid formation	Not focused	110
2021	1	26	10× Genomics	3301 total	Hypospermatogenesis?**	Idiopathic/primary hypogonadism	Increased	Small	Not focused	1.Lower mature LCs in NOA patients but more LCs experiencing cell division/differentiation 2.Identifying DEGs of immature LCs	78
2021	3	37/37/41	10× Genomics	3880 total	SCOS	Idiopathic	Increased	Small*	Not focused	1.Finding the special transcriptional characteristics of somatic cells in SCOS, including immaturity of LC (*p16*/*CDKN2A*+), senescence of somatic cells, pro-inflammatory status of the microenvironment 2.The circulatory inflammatory markers such as CCL4 and CCL5 were enhanced in SCOS patients 3.Reporting a high level of DNA damage in SCOS LC and a genetic mutation in *LMNA* of SCOS testes	88
2022	1	31	Slingleron GEXSCOPE^TM^	1212 total	SCOS	Idiopathic	Increased	Small	Not focused	1.Identifying the special transcriptomic pattern of NOA Sertoli cells, including the decrease of cell junction genes (*SYNE2*, *ATP2B1*, *MTDH*) and increase of *FATE1*	56

*Confirmed by checking with the first author of original articles. **Successful testicular sperm retrieval but only few sperm were found, which inferring the pathology of hypospermatogenesis. Note: FSH, follicle-stimulating hormone; SCOS, Sertoli cell-only syndrome; DEGs, differential expressed genes. Note2: NOA phenotype patients with clear evidence of chromosomal abnormality (such as KS or Y chromosome microdeletion) were excluded from this table and will be specially discussed in a separate part.

**Table 4:** Studies only with reanalysis of previously published scRNA-seq data of human testicular samples

Authors	Year	Country*	Human testicular scRNA-seq data type	Other key public/published datasets/databases used**	Main findings	Important experiments/clinical studies and original sequencing data	Reference
Gomes Fernandes et al.	2018	Netherlands	Prenatal	1.ScRNA-seq data of gonadal cells from fetal ovaries (GEO)	1.PIWIL1 was barely expressed in fetal testes but located paranuclearly as a single large dense satellite-like body in oocytes 2.PIWIL2 was detected in the cytoplasm of both female and male germ cells but shrank in intermitochondrial cement in oocytes in primordial follicles 3.PIWIL3 was absent in germ cells of both genders 4.PIWIL4 was found in the cytoplasm of germ cells and localized in intermitochondrial cement of both oocytes in primordial follicles and spermatogonia	1.Immunofluorescence (IF) staining of fetal testes/ovary, mouse testes and adult ovary 2.Fluorescence-activated cell sorting (FACS) analysis of fetal testicular or ovarian cells	187
Pont et al.	2019	France	Normal	1.ScRNA-seq data of other human tissue/cells (GEO/ArrayExpress/ 10× Genomics website 188)	1.Developing a method (Single Cell Signature Explorer) for gene signature scoring and its visualization at a single-cell resolution (https://sites.google.com/site/fredsoftwares/products/single-cell-signature-explorer)	No	174
Reznik et al.	2019	USA	Prenatal	1.Human transposable element consensus sequences from GIRI Repbase dataset 189	1.Drawing the dynamic maps of PIWI proteins' expression and identifying transposon-derived piRNAs of fetal testes across development, and detecting an active piRNA pathway and transposable elements repression 2.Finding pre-pachytene piRNAs amplified in human fetal testes, which were mostly derived from transposable elements especially long interspersed element type 1 family (L1) 3.Finding L1-ORF1p (coded by L1 gene open reading frame 1) were highly expressed at mid-gestation and decreased in germ cells along with the enhancement of piRNAs, H3K9me3 and HIWI2 nuclear localization 4.Finding the evidence that L1 expression is heterogeneous, in other words, a subset of L1-expressing fetal germ cells utilized the PIWI-piRNA pathway to cause epigenetic silencing of L1 via H3K9me3, but the rest cells were L1-resistant	1.Human fetal testes-based experiments including IF staining and small RNA-seq, etc. 2.Xenograft model (mice)-based experiments including model construction (implanting human fetal testes into immunocompromised mice) and xenograft samples IF staining, etc.	190
Wang et al.	2020	USA	OB/Normal	No	1.Revealing the expression of *ACE2* (SARS-CoV-2 target) in SPGs, LCs and SCs	No	145
Pan et al.	2020	China	Normal	No	1.SARS-CoV-2 was not detected in the semen of patients recovering from COVID-19 2.There was barely any co-expression of *ACE2* and *TMPRSS2* based on scRNA-seq data	1.Collecting semen samples from COVID-19-recovered male Chinse patients (along with clinical information) and do RT-qPCR of SARS-CoV-2 in semen samples	146
Xia et al.	2020	China	OB	No	1.Finding endosialin as a specific marker of human stem Leydig cells 2.Finding that endosialin^+^ cells were with obvious proliferation (self-renewal) and differentiation (to testosterone-producing LCs) ability *in vitro* 3. Finding that transplanted human endosialin^+^ cells could localize in the interstitial region of murine testes and gain proliferation and differentiation ability *in vivo*	1.Cell experiments of human testicular endosialin^+^ cells including cell isolation, cell culture, cell proliferation assay, *in vitro* cell differentiation, RT-qPCR, lentiviral vector infection, enzyme-linked immunosorbent assay (ELISA), flow cytometry, IF staining, etc. 2.IF staining of human and mouse testicular tissue 3.*In vivo* experiments by transplanting human endosialin^+^ cells in mouse testes	191
Ceyhan et al.	2020	USA	Normal	1.Gene microarray data of testes from cryptorchid/infertile or normal persons (ArrayExpress)	1.*INPP4B* is predominantly expressed in post-meiotic spermatogenic cells of both human and mice testes 2.*Inpp4b*-/- male mice were with smaller testes and fewer sperm, which could be worsened by aging and high-fat diet, and decreased steroidogenic enzymes and LH receptor gene were also found 3.*Inpp4b*-/- male mice had decreased elongated spermatids with increased diploid cells in testes 4.Finding enhanced apoptosis rate in spermatogenic cells and reduced meiotic marker γH2A.X expression in *Inpp4b*-/- mice testes	1.Animal experiments including the establishment of gene knockout mice models, different feeding, sperm count and hormone measurement 2.RT-qPCR, immunohistochemical (IHC) staining, Western blotting (WB), propidium iodide staining and flow cytometry of testicular tissue of mice	170
Zhou et al.	2020	China	OB	1.ScRNA-seq data of multi organs/cells (GEO/HPA) 2. IHC staining of ACE2/ TMPRSS2/Furin proteins from HPA 192, and bulk RNA expression level of these three genes from HPA 192 and GTEx 193	1.Ranking the organs and cells which were easier to be harmed by SARS-CoV-2 and finding the candidates with high risk including lung AT2 cells and macrophages, cardiomyocytes, stromal cells of the adrenal gland, the testis, the ovary and the thyroid 2.Finding that an acid condition could inhibit the activity of SARS-CoV-2 pseudovirus, which indicated the defensive role of the stomach against SARS-CoV-2 infection	1.Infecting 293T-ACE2 and Hela-ACE2 cell lines with pseudovirus of SARS-CoV-2 pretreated with pH of 1.0, 2.0, 4.0, or 7.0 and luciferase assay of infected cells (24h or 48h)	147
Jiang et al.	2020	USA/China	OB	1.ScRNA-seq data of mouse testes (GEO)	1.Finding the total opposite survival status of *Trf2*^Fl/Fl^;*SFTPC*-Cre^Yo^ (100%viability) and *Trf2*^Fl/Fl^;*SFTPC*-Cre^Blh^ (100%lethality), which was driven by heterogeneity in embryonic expression of those two transgenes 2.Finding the activity of both *SFTPC*-cre lines in the male germline, indicating premeioticly expressed Cre recombinase 3. Finding that *SFTPC* only expressed in late spermatogenesis of human and were absent in murine spermatogenesis	1.Animal experiments based on *Trf2* floxed, *SFTPC*-cre^Yo,^ *SFTPC*-cre^Blh^ and *Rosa26*^mT/mG^ mice and their crosses 2.Hematoxylin-Eosin (HE), IHC and IF staining of mouse testes	171
Winge et al.	2020	Denmark	Normal/OB/KS	1.Bulk RNA-seq or gene/methylation microarray data of testis/blood/cultured lymphocytes of KS patients (GEO/EGA 194, etc.)	1.Reviewing the histopathological changes of KS patients (fetal, underage and adult), such as the loss of germ cells starting from fetal time, hyalinized tubules, loss of SCs and LCs hyperplasia 2.Identifying a series of genes (by comparing analyses on DEGs/DMRs among KS studies) which is related to escape X-inactivation and might lead to the pathogenesis of KS 3.Revealing that SCs might be the most altered cells in terms of transcriptional changes	1.HE staining of testes from normal karyotype and KS patients	126
Lau et al.	2020	Singapore	Normal/OB	1.ScRNA-seq data of mouse testes (ArrayExpress)	1.Drawing the map of spermatogenesis of cynomolgus macaque 2.Identifying different subtypes of SPG, revealing their marker genes and detecting the self-renewal versus differentiation dynamics of SPG in cynomolgus macaque 3.Showing both the similar and different genes expressed among human, mouse and cynomolgus macaque during the spermatogenic process and finding the conservatism of meiotic sex chromosome inactivation among three species	1.Original scRNA-seq data of the cynomolgus macaque testis 2.HE, IHC and IF staining on testes of cynomolgus macaque	167
Shen et al.	2020	China	Normal/OB/NOA	1.Interaction network data of *ACE2* from GeneCards 195	1.Detecting the expression of *ACE2* (SARS-CoV-2 target) in germ cells and somatic cells 2. Detecting the higher positive rate of *ACE2* in infertile men than in normal persons	No	148
Chen et al.	2020	China	Normal	1.In-frame indels in tandem repeat regions from the UCSC genome browser database 196 2.Gene information from Consensus Coding Sequence (CCDS) database 197, and gene variants from databases including 1000 Genomes Project 198, ESP6500siv2 199 and gnomAD 200 3.Gene expression data from GTEx and HPA 4. Mouse phenotype information from the MGI database	1. Identifying six pathogenic/likely pathogenic variants and four unknown-significance variants (VUS) in genes that could cause NOA or severe oligospermia (SO) 2. Reporting 20 new genes which might be related to NOA/SO, and five of them (*BRDT*, *CHD5*, *MCM9*, *MLH3* and *ZFX*) were considered as strong candidates based on testicular single-cell data and previously reported murine models	1. Whole-exome sequencing of 314 NOA or severe oligospermic Chinese patients and sanger sequencing	139
Hikmet et al.	2020	Sweden	Normal	1.ScRNA-seq data of other tissues (GEO/ COVID-19 Cell Atlas 201) 2.RNA expression data of testes and other organs from FANTOM5, GTEx and HPA 3.Protein expression data of testes and other organs from PaxDB 202 and ProteomicsDB 203	1.Comparing the expression level of *ACE2* (SARS-CoV-2 target) among different tissues and finding the high expression of *ACE2* mRNA and protein in the testis, especially LCs and SCs	1.IHC staining of ACE2 in the human testis and other organs 2.WB of ACE2 in human lung/tonsil/kidney/colon	149
Ren et al.	2020	China	OB	1.Clinical and epidemiological data of COVID-19 patients from public databases 204, 205 2.Transcriptional data and microarray of testes and other organs from FANTOM5 and GTEx and GEO database 3.Transcriptional data of normal and testicular cancer and other cancers from TCGA 4.IHC staining data of testes and other organs from HPA 5.ScRNA-seq data of kidney from KIT [206, 207]and HCL 183 databases	1.Identifying the high expression of *ACE2* and *TMPRSS2* in kidney with chronic renal diseases or diabetic nephropathy 2.Identifying enrichment of *ACE2* in testicular germ cells and renal proximal tubules 3.Identifying the concentration of pro-inflammatory cytokines (like *IL6ST*) in testicular EC, macrophages, SSC as well as renal endothelial cells	No	150
Stanley et al.	2020	UK	Normal	1.ScRNA-seq data of cynomolgus monkeys' ovary (GEO) 2.Transcriptional data and protein expression level of testes and other organs from HPA and HPM database 208 3.Transcriptional data of EFO-21, AN3-Ca and BEWO cell lines from human cell atlas 209	1.Reporting the lack of co-expression of *ACE2* and *TMPRSS2* among testicular cells and ovarian somatic cells and the expression of both in a subpopulation of oocytes 2.Showing the wide expression of *ACE2* with a lack of expression of *TMPRSS2* in human cumulus cells 3.Suggesting the unlikeliness of long-term impact on human reproductive glands caused by SARS-CoV-2	1.Original RNA-seq of human cumulus cells from nine individuals	151
Qi et al.	2021	China	Normal	1.ScRNA-seq data of other human organs (GEO/Tissue Stability Cell Atlas 210)	1.Investigating *ACE2* and *TMPRSS2* expression in different cell types of 31 organs and finding gall bladder and fallopian tube might be harmed by SARS-CoV-2 infection 2.Recognizing human nose, heart, small intestine, large intestine, esophagus, brain, testis, and kidney as “high-risk organs” due to high expression levels of *ACE2* and *TMPRSS2*	No	152
Soraggi et al.	2020	Denmark	Normal/OB	No	1.Reviewing the genetic causes of spermatogenic dysfunction 2.Reviewing common data-processing procedures and visualization methods of scRNA-seq data 3.Doing integrated analysis of three independent testicular scRNA-seq data and analysis of some NOA-related genes 4.Building up an interactive website (https://testis.cells.ucsc.edu/) of integrated testicular scRNA-seq data	1.HE staining of the human testis (OA and NOA)	177
Zheng et al.	2021	China	Normal	1.Microarray of NOA testes and related controls (GEO) 2.IHC staining of testes from HPA	1.Showing the largest group of immune cells in the normal testis was macrophages which kept an anti-inflammatory status 2.Reporting and validating a negative correlation of both M1/M2 macrophages with testicular Johnsen scores and an enhanced number of both M1/M2 macrophages in NOA	1.IHC staining (CD163/CD68/CD86/INOS) of the human testis (NOA and normal spermatogenesis)	142
Olivieri et al.	2021	USA	OB	1.ScRNA-seq data of various tissues from human (Tabula Sapiens 211), mouse lemur (Tabula Microcebus 212) and mouse (Tabula Muris 213/GEO)	1.Drawing the RNA splicing profiles of 12 human tissues using the SpliZ method and showing that splicing is regulated cell-type-specifically 2.Finding a number of cell-type-specifically spliced genes based on 10×Chromium scRNA-seq data, including *MYL6* and *RPS24*, which were validated by FISH, Smart-seq2 and single-cell RT-PCR 3.Revealing conserved regulated splicing in spermatogenesis among human, mouse and mouse lemur (such as *CEP112* and *SPTY2D1OS*) 4.Identifying subpopulations of monocytes (using SpliZ) with subpopulation-specific splicing of an ultraconserved exon of *SAT1*	1.Fluorescence *in situ* hybridization (FISH) tests on human lung/muscle tissue and isolated muscle cells 2.Sanger sequencing and single-cell RT-PCR	165
He et al.	2021	China	OB	1.Gene microarray data of testes from NOA/OA patients or pooled controls (GEO)	1.Identifying three hub genes including *C22orf23*, *TSACC*, and *TTC25* which were related to spermatogenesis 2.Finding that *TTC25* kept a low level through spermatogenesis, and *C22orf23* had two peaks (one in diff_SPGs and one in late primary spermatocytes), while *TSACC* had an increasing trend during spermatogenesis	No	143
Hadziselimovic et al.	2021	Switzerland	Normal/OB/NOA	1.Bulk RNA expression data of human testes and other tissues from GTEx 2. Genome-wide annotations of regulatory sites information in the SwissRegulon dataset 214	1.Finding *NHLH2* to be decreased in pre-pubertal high infertility risk patients and reversed by GnRHa treatment	No	215
Zhang et al.	2021	China	OB	1.Bulk RNA expression of *ACE2* from testes and other organs based on GEPIA2 and Expression Atlas 216 2. IHC staining of ACE2 on human testes from HPA	1.Finding the high expression level of *ACE2* mRNA in the testis compared with other organs 2.Finding that Leydig cells might be the target of SARS-CoV-2 and could be infected by pseudovirus SARS-CoV-2 in mice 3.Recovered COVID-19 patients had significantly lower testosterone levels than healthy controls	1.Animal experiments including intratesticular injection with pseudovirus SARS-CoV-2 in mice (including infection detecting by Immunofluorescence), and IHC staining of ACE2 on mice testes 2.Testing sexual hormone levels in patients recovered from COVID-19 and controls	153
Yang et al.	2021	China	OB/NOA	No	1.*ELAVL2* was highly expressed in the testis of human and mice but decreased in NOA testes 2.ELAVL2 was predominantly located in SSCs of human and mice 3.ELAVL2 could facilitate the proliferation and reduce apoptosis of C18-4 and TCam-2 cell lines by sensitizing ERK and AKT pathways 4.ELAVL2 as an RNA-binding protein could bind mRNAs that were important in regulating SSC survival and proliferation and enhance their protein level post-transcriptionally 5.ELAVL2 could interact with DAZL in human and mouse testes	1.Cell experiments based on primary testicular cells or testicular cell lines including cell culturing, IF staining, RT-qPCR, WB, Cell Counting Kit-8 (CCK-8) and EdU incorporation assay, lentivirus infection, Annexin-V/PI staining with flow cytometry, TdT-mediated dUTP Nick-End Labeling (TUNEL) Assay and co-IP, etc. 2.Human/mouse tissue-based experiments including IF staining, RT-qPCR, WB, Co-Immunoprecipitation (Co-IP), RNA Binding Protein Immunoprecipitation (RIP) and mass spectrometry, etc.	160
Han et al.	2021	China	Normal/NOA	1.Gene microarray of NOA/control testes (GEO)	1.Revealing the DEGs and associated signal pathways in NOA patients compared to control 2.Identifying *CHD5* and *SPTBN2* as potential biomarkers of NOA pathogenesis and showing its obvious downregulation in NOA testes among various testicular cell types	No	144
Salehi et al.	2021	Iran	Normal/OB	No	1.Identifying ten bridge genes (highest betweenness centrality) among different stages of spermatogenesis, including *DNAJC5B*, *C1orf194*, *HSP90AB1*,* BST2*, *EEF1A1*, *CRISP2*, *PTMS*, *NFKBIA*, *CDKN3*, and *HLA-DRA*	No	217
Fan et al.	2021	Netherlands	Normal/OB	1.ScRNA-seq data of fetal female gonads (GEO) 2.Gene lists related to male and female infertility from the DisGeNET v6.0 database 218	1.Drawing the maps of molecular signatures (marker genes, DEGs among subclusters, dynamic expression changes of functional genes such as cytoskeleton-associated genes, etc.) during female meiotic prophase I stages 2.Revealing both conserved and sex-distinctive transcriptional features as well as DNA methylational regulation during meiotic prophase I stages between female and male 3.Revealing a momentary increase of X-linked expression during female pachytene (which is opposite to meiotic sex chromosome inactivation of males) and revealing that it was due to a lower turnover or higher stability of X-linked genes rather than enhanced X-linked transcription	1.IF staining on fetal ovaries/testes and adult ovaries/testes 2.RNA/DNA FISH on fetal ovaries and RNA FISH on adult testes	172
Persio et al.	2021	Germany	OB/Cryptozoospermia	1.Whole-genome bisulfite sequencing (WGBS) of sperm/ embryonal stem cells/SSC/ primordial germ cells (GEO/ENA 219)	1.Finding no obvious difference between control and cryptozoospermia patients in terms of whole genome levels' or imprinted regions' DNA methylation 2.Identifying a series of differentially methylated regions (DMRs) (in cryptozoospermia) and their related genes, most of which were hypermethylated 3.Further recognizing 13 DMR-associated (most were hypermethylated DMRs) genes (*BBS5*, *C2orf92*, *C10orf120*, *CFAP299*, *DHX16*, *MTCP1*, *PACRG*, *PPP1R36*, *PLAC8L1*, PPP3CC, *SPAG16*, *TCP10L2*, *ZFAND4*), which were differentially expressed (most were downregulated) in meiosis and/or spermiogenesis of cryptozoospermia patients 4.Finding that four genes (*C2orf92*, *C10orf120*, *PPP3CC*, and *PPP1R36*) had hypermethylated promoters in cryptozoospermia while the rest might have methylated germ cell-specific enhancers	1.Periodic Acid-Schiff (PAS) staining of human testes (control/ cryptozoospermia) 2.Isolation and culturing of primary testicular cells and ploidy analysis 3.Targeted deep bisulfite sequencing and WGBS of testicular cells (control/cryptozoospermia)	220
Martin-Inaraja et al.	2021	Spain	Prenatal	No	1.Evaluating the effects of two different culture media (Shinohara-medium and Zhou-medium) and four substrates (laminin, gelatin, vitronectin and matrigel) on culturing human fetal germ cells and finding the huge differences of hFGC numbers in different culture environment 2.Finding that the Shinohara-medium with gelatin-coated substrate could lead to the highest number of hFGCs (10% in day 6) while vitronectin-coated substrate could lead to similar hFGCs number (day 6) in two media	1.Isolation, FACS analysis and culturing of male human fetal germ cells from fetal testes 2.IF staining of human fetal testes and cultured human fetal germ cells	141
Hardy et al.	2021	USA	Normal	1.Mouse model phenotypes in MGI database 2. Gene expression data of testes using GTEx, Ace-View 221 and BioGPS 222-224 3. SprT-like domain 3D modeling and intrici disordered region motif search of *GCNA* via several online database/tools such as UniProt 225, HHpred^226^, MODELLER^227^ , MetaDisorder^228^, etc.	1.Identifying seven potential pathogenic variants (NOA or cryptozoospermia) of *GCNA*, such as p.Ala115ProfsTer7(which caused early frameshift), p.Ser659Trp/p.Arg664Cys(located in SprT-like domain), and variants such as p.Ser295Pro which were located in predicted consensus IDR motif 2.Finding the expression of *GCNA* throughout spermatogenesis, especially in differentiating SPGs	1.Orignial whole genome sequencing (WGS) of 2225 NOA patients (with clinical information analyses) from multi-centers 2.PAS and IF staining of human testicular tissue (normal/*GCNA* variants)	229
Zhou et al.	2021	China	NOA	1.Gene microarray of NOA/OA testes (GEO)	1.Building up a five-gene based random forest diagnosis model (*CCT8*/*CDC6*/*PSMD1/RPS4X*/*RPL36A*) for NOA and OA 2.Validated those five genes' expression and the efficacy of the random forest model in local cohort	1.IHC staining of RPS4X on human testes (20 OA and 20 NOA samples) 2.RT-qPCR on seminal plasma (20 OA and 20 NOA) of five genes (*CCT8*, *CDC6*, *PSMD1*, *RPL36A*,* RPS4X*) 3.Analysis of clinical (age, sex hormones) and pathological (Johnsen scores) parameters of the above-mentioned 40 patients	161
Cai et al.	2021	China	Normal/Infant	1.Transcriptional data of normal testes from GTEx database	1.Analyzing the expression of of *ACE2* (SARS-CoV-2 target) in both adult testes and infant testes 2.Validating the high expression of ACE2 protein in testes	IHC staining of ACE2 on human normal testes	154
Wu et al.	2022	China	Normal	No	1.Drawing the original single-cell Assay for transposase-accessible chromatin sequencing (scATAC-seq) based map of human spermatogenesis (including accessibility of chromosomes, TF-binding sites and motifs with high frequencies during spermatogenesis, etc.) and showing scATAC-seq's advantages over scRNA-seq 2.Identifying two spermatogenically functional genes including* TLE3* (exclusively expressed in diff_SPGs) and *PFN4* (relevant to actin cytoskeletal organization during meiosis) and finding the motifs (such as *CTCF*, *CTCT*, etc) with high accessibility which were enriched in the upstream of these two genes	1.Original scATAC-seq of human normal testes	230
Tang et al.	2022	China	OB/NOA	No	1.Showing a significant increase of LC and macrophages in iNOA patients' testes 2.Identifying of LC-specific transcription factors (TFs) (including *LHX9*, *KLF8*, *KLF4*, *ARID5B* and *RXRG*) in NOA, and macrophages-specific TFs (such as *POU2F2*, *SPIB*, *IRF5*, *CEBPA*, *ELK4* and *KLF6*) in NOA, which might be related to the function of LC and macrophages and finally lead to impaired spermatogenesis	No	164
Zhang et al.	2022	China	Juvenile/NOA	1.Gene microarray of mouse testicular Sertoli cells exposed to Bisphenol A	1.Finding the proliferation and maturation of spermatogenic cells caused by Bisphenol A, which might be triggered by secretory proteins from SC 2.Finding that Bisphenol A might be able to harm SC by dysreguating its secretory proteins, and might be a potential cytotoxic factor related to NOA	No	163
Rengaraj et al.	2022	South Korea	Prenatal	No	1.Establishing a transgenic chicken model (*DAZL::GFP*) with GFP expressing but not changing the expression of endogenous DAZL 2.Identifying four male-specific and five female-specific stages during the development of chicken germ cells, and revealing the paths as well as their related transcriptional patterns of chicken germ cells' stages transition (one path in male and two paths including meiosis and apoptosis in female) 3.Revealing both conserved and species-specific transcriptional patterns between human and chicken germ cell development	1.Isolation (using MACS) and culturing of chicken primordial germ cells from male gonads 2.Building up *DAZL::GFP* PGCs and *DAZL::GFP* transgenic chickens 3.Validation of the transgenic PGCs and chickens by WB, RT-qPCR and IF staining, etc. 4.Original scRNA-seq of the *DAZL::GFP* germ cells collected from embryos or embryonic gonads (both male and female) at different ages	166
Wu et al.	2022	China/USA	Normal/OB/NOA	No	1.Reviewing the role of laminin and collagen chains (bioactive peptides) in regulating rodents' spermatogenesis 2.Showing the different expression level of important genes encoding laminin and collagen chains or basement membrane proteins among normal, OA and NOA human testes, suggesting their supporting roles in human spermatogenesis	1.HE staining of human testes with complete spermatogenesis 2.IF staining of α-tubulin and F-actin on human SC	137
He et al.	2022	China	Normal	1.ScRNA-seq data of human head and neck carcinoma (GEO) 2. ScRNA-seq data (PLIN2 expression level) of various organs (lung/pancreas/prostate) from HPA 3.Data of tumor infiltrating immune cell markers of oral squamous cell carcinoma (OSCC) from TIMER 231 4.Gene expression data of head and neck squamous cell cancer in cBioPortal 232, 233	1.*PLIN2* is highly expressed in macrophages in human testes and other organs (lung, pancreas and prostate) and CD68+ tumor-associated macrophages of OSCC stroma 2.OSCC patients with higher CD68+ TAM-derived PLIN2 expression were with advanced stages, more malignant phenotypes and poorer prognosis 3.An immune suppressed features (low CD8+ T cells/high CD68+ TAMs and FOXP3+ Tregs with enhanced immune checkpoints) were observed in OSCC patients with high PLIN2 expression	1.IHC, IF and Oil Red O staining of human oral squamous cell carcinoma tissues 2.Clinicopathological data analysis and survival analysis of OSCC patients	138
Zhang et al.	2022	China	Juvenile/Normal	No	1.Identifying five SC subtypes, five LC subtypes and four PTM subtypes in pigs during somatic development 2.Identifying *PRND* as a new maker of SCs 3.Revealing the high conservatism of somatic cell development between human and pigs (except for LCs) and screening some transcription factors involved in the development of somatic cells of pigs and human	1.Origial scRNA-seq of pig testes at different ages 2.HE and IF staining (SOX9/PCNA/AMH/ STAR/ACTA2/PRND) of pig testes	169
Zhou et al.	2022	China	NOA	1.Gene microarray data of OA/NOA and pooled control testes (GEO) 2. Gene symbols of transcription factors (TFs) from Human Transcription Factor database 234	1.Building up a three-gene based random forest diagnosis model (*ETV2*/*TBX2*/*ZNF689*) for NOA 2.Validated those three genes' expression and the efficacy of the random forest model in local cohort	1.RT-qPCR (*ETV2*, *TBX2* and *ZNF689*) of seminal plasma from 20 NOA and 20 OA (with normal spermatogenesis) 2.IHC staining of ETV2/TBX2/ZNF689 on testicular samples of the above-mentioned 40 patients 3.Analysis of clinical (age, FSH, LH, T) and pathological (Johnsen scores) parameters of the above-mentioned 40 patients	140
Chitiashvili et al.	2022	USA	Prenatal	1.ScRNA-seq data of fetal ovaries, fetal germ cells and day 4 aggregate cells (GEO)	1.Showing that *FGFR3* were enriched in primordial germ cells (PGCs) of both ovaries and testes, but decreased with the appearance of primordial oocytes 2.Based on the evidence from fluorescence-activated cell sorting and scRNA, finding that FGFR3 could work as an *in vitro* biomarker to enrich FGFR3-positive PGCs from the human embryonic and fetal ovaries	1.IF staining of fetal ovaries and day 4 aggregate cells differentiated from human embryonic stem cells (hESC) 2.Fluorescence activated cell sorting of fetal ovarian cells 3.Culturing of hESC and differentiating into primordial germ cell-like cells (PGCLC) 4.Original scRNA-seq of FGFR3-sorted fetal ovarian cells and PGCLC	235
Guo et al.	2022	China	Normal	1.Clinicopathological, transcriptional, DNA-methylation data and copy number of TCGA testicular germ cell tumor (TGCT) from UCSC XENA database 236 and microarray data of TGCT (GEO) 2. Expression levels of *RFPL3S* in different tumors from GEPIA2 database, and immune data of TGCT (immune scores, tumor infiltrating immune cells, immune, and reactions to immune therapy, etc.) from Sangerbox 237, GSCA 238 and TIDE databases 239.	1.Revealing that long non-coding RNA *RFPL3S* might be a tumor suppressor gene in TGCT (with low expression, hypermethylation and low copy number in TGCT, and was negatively associated with the stage of TGCT) and validating this phenomenon by cell experiments 2.Showing that *RFPL3S* was mainly expressed in testicular germ cells and were positively associated with the infiltration of immune-activating cells and immunotherapy benefits, while negatively related to immunosuppressive cells	1.Cell experiments including transfection of si-*RFPL3S*, RT-qPCR of *RFPL3S*, transwell assay and CCK-8 assay based on NCCIT and Tcam-2 cell lines	136
Tian et al.	2022	China	Normal	1.ScRNA-seq data of testes of mice and cynomolgus macaque	1.Constructing the profile of spermatogenesis of Mongolia sheep using scRNA-seq data 2.Comparing the spermatogenic process of human, mice, sheep and cynomolgus macaque and finding the conserved meiotic sex chromosome inactivation and genetic dynamics of spermatogenesis among four species 3.Revealing the high conservatism of spermatogenic cell development, gene expression and testicular cell-cell communication signaling between human and sheep	1.Original scRNA-seq of the Mongolia sheep testis 2.IF staining (DMRT1/ TEX101/KLF5/WWTR1) on the Mongolia sheep testis	168
Yang et al.	2022	China	OB	1.Exome Aggregation Consortium (ExAC) data 240 and 1000 Genomes Project 198 2. Known pathogenic genes of murine azoospermia from MGI database 3. Human testes enriched genes from HPA database	1.Identifying a new copy number variation (CNV) (seq [GRCh37] del(19) (19q13.33) chr19: g.49894043-49903011del) and a heterozygous loss of function variant (NM_144688: c.979_980del: p.R327Sfs*21) in* KASH5* gene of a NOA patient (with meiotic arrest pathology) and his sister 2.Reporting the expression of *KASH5* in human testes (mostly in SPCs) and finding the similar expression pattern of it between human and murine testes 3.Validating the similar phenotype (NOA) using *Kash5*-null mouse model	1.Original scRNA-seq data of mouse testes 2.HE and IF staining on human (OA and meiotic arrest) testes 3.Whole-exome sequencing, PCR, RT-qPCR, sanger sequencing, CNV array together with clinical data analysis of an NOA (meiotic arrest) patient with bi-allelic variants in *KASH5* and his family members 4.Establishing *Kash5*-heterozygotes and *Kash5*-null mice model (together with wide type mice) and HE/IF/TUNEL staining & WB of mice testes 5.Meiotic chromosomal spread experiments on human and murine testes	135
He et al.	2022	China	OB/Neonatal/KS	1.Bulk RNA-seq data of adult/fetal testes from Klinefelter syndrome (KS) patients with adult/fetal normal controls (GEO)	1.Identifying four un-reported hub genes (*KIF2C*, *MRPS2*, *RPS15* and *TSFM*) which might be functional in KS 2.Finding that *KIF2C* showed an increasing trend in the development of spermatogenetic cells and might be strongly relevant to germ cell development of KS patients	No	127
Calonga-Solís et al.	2022	Germany	Fetal/Neonatal/Infant/Juvenile/OB	1.ScRNA-seq data of prenatal ovaries 2.Binding site information in GeneHancer database 241	1.Detailed case report of a patient with de novo stop-gain variant in *MYRF* (p.Q838*), which was associated with the patient's Scimitar syndrome, 46,XY partial gonadal dysgenesis and severe hyperopia 2.Reporting the expression status of *MYRF* using scRNA-seq data of human gonads at different ages, and finding a high expression of *MYRF* in subsets of coelomic epithelium cells for both male and female 3.Suggesting that *MYRF* might involve in early development of gonad and heart by upregulating *CITED2*	1.Detailed case report of a patient with Scimitar syndrome, 46,XY partial gonadal dysgenesis and severe hyperopia 2.Trio-WGS of the patient's family (mother, father and affected child)	242
Stow et al.	2022	USA	Normal	1.ScRNA-seq data of mouse testes and MCF7 cell line (NCBI Sequence Read Archive 243)	1.Developing and testing a new method named SCIFER (including technical parameters needed), to quantify expression of long interspersed element-1 at the single-locus resolution in scRNA-Seq datasets 2.Pointing out that unsupervised analysis of L1 expression in single cells exponentially inflates the levels of L1 expression and the number of expressed L1 loci 3.Finding that mouse round spermatids and human SPGs, SPCs, and round Spermatids had the highest levels of L1 mRNA	1.Original scRNA-seq of MCF7 and HEK293-FRT-LacZeo combined cells 2.Original bulk RNA-seq of human testicular bulk RNA samples (commercial)	175
Luo et al.	2022	China	Normal/OB	No	1.Recognizing the upstream trend of *FOXP4* with the development of SSC, and showing it as a marker of a subset of SPG that had stem cell features 2.Proving that the inhibition of *FOXP4* could significantly repress SSC's proliferation and enhance its apoptosis 3.Showing that FOXP4 were significantly downregulated in human testes with spermatogenic dysfunction, especially in those with severe pathological pattern	1.Tissue based experiments including HE and IHC (FOXP4)/ IF staining (FOXP4/GRFA1/UCHL/PCNA/KIT) and WB (FOXP4) of testicular samples from OA and NOA patients 2.Cell experiments including cuturing of human SCC cell line, si-*FOXP4* transfection, RT-qPCR (*FOXP4*), CCK-8 assay, EdU assay, Flow cytometry with Annexin V-APC/PI staining, TUNEL assay and WB (FOXP4/PCNA)	162

*Country information was based on the institutional information of the first author **To be more brief, the references of some databases employed by multi-studies were listed here, including GEO (Gene Expression Omnibus) 244, ArrayExpress^245^, HPA(Human Protein Atlas 192, 246, GTEx (Genotype-Tissue Expression) 193, FANTOM5 (Function Annotation of The Mammalian Genome) 247, TCGA (The Cancer Genome Atlas) 248, GEPIA2 (Gene Expression Profiling Interactive Analysis) 249 and MGI (Mouse Genome Informatics) 250 databases.

**Table 5:** Popular interaction-friendly online portals/websites containing scRNA-seq data of human testicular samples

Author	Year	Country	Portal name	Website	Testicular data type	gonad-specific portal?	Reference
Guo et al.	2018	USA	UCSC cell browser-(ID)adult-testis	https://cells.ucsc.edu/?ds=adult-testis	Normal	Yes	31
Franzén et al.	2019	Sweden	PanglaoDB	https://panglaodb.se/	Normal/Underage	No	251
Darde et al.	2019	France	ReproGenomics Viewer	https://rgv.genouest.org/	Normal/OB/NOA/Prenatal	Yes*	180
Soraggi et al.	2020	Denmark	UCSC cell browser-(ID)testis	https://cells.ucsc.edu/?ds=testis	Normal/OB	Yes	177
Singh et al.	2020	USA	UCSC cell browser-(ID) scarface	https://cells.ucsc.edu/?ds=scarface	OB	No	252
Han et al.	2020	China	Human cell landscape (HCL)	http://bis.zju.edu.cn/HCL/	Normal/Prenatal	No	183
Chen/Chitiashvili/Guo et al.	2020	USA	Human germline developmental atlas	https://germline.mcdb.ucla.edu/	Prenatal	Yes	79, 178, 179
Cairns Lab/Guo et al.	2020	USA	Human Testis Atlas	https://humantestisatlas.shinyapps.io/humantestisatlas1/	Normal/Underage	Yes	31, 72
Karlsson et al.	2021	Sweden	The human protein atlas (HPA)	https://www.proteinatlas.org/humanproteome/single+cell+type	Normal	No	246
Zheng et al.	2021	China	ColorCells	https://rna.sysu.edu.cn/colorcells/	Normal/OB/NOA	No	253
Mahyari et al.	2021	USA	Human infertility single-cell transcription atlas (HISTA)	https://conradlab.shinyapps.io/HISTA/	Normal/OB/KS/NOA/Ejaculatory dysfunction/Underage	Yes	78
Li et al.	2022	Singapore	Deeply integrated human single-Cell omics data (DISCO)	https://www.immunesinglecell.org/	Normal/Prenatal/Underage/OB/NOA/KS	No	254
Chan Zuckerberg Initiative	-	USA	CZ CELLxGENE Discover	https://cellxgene.cziscience.com/	Prenatal	No	255

* In terms of scRNA-seq data

## Abbreviations

ScRNA-seq: single-cell RNA sequencing
GEO: Gene Expression Omnibus
OA: obstructive azoospermia
TESE: testicular sperm extraction
OB: obstruction
FACS: fluorescence-activated cell sorting
MACS: magnetic activated cell sorting
SPG: spermatogonia
SPC: spermatocyte
SSC: spermatogonial stem cell
diff_SPG: differentiating SPG
diffed_SPG: differentiated SPG
L.SPC: leptotene SPC
Z.SPC: zygotene SPC
P.SPC: pachytene SPC
D.SPC: diplotene SPC
SC: Sertoli cell
LC: Leydig cell
NOA: non-obstructive azoospermia
SCOS: Sertoli cell-only syndrome
PGC: primordial germ cell
FGC: fetal germ cell
CE: coelomic epithelium
PLCs: progenitor LCs
ILCs: immature LCs
MLCs: mature LCs
PGCLs: primordial germ cells-like cells
iNOA: idiopathic NOA
OAT: oligo-astheno-teratozoospermia
mTESE: microdissection TESE
ICSI: intracytoplasmic sperm injection
KS: Klinefelter Syndrome
AZFa: azoospermia factor a
AZFb: azoospermia factor b
AZFc: azoospermia factor c
IHC: immunohistochemistry
IF: immunofluorescence
WGS: whole genome sequencing
COVID-19: corona virus disease 2019
L1: long interspersed element-1
UMAP: Uniform manifold approximation and projection
t-SNE: t-distributed stochastic neighbor embedding
FSH: follicle-stimulating hormone
DEGs: differential expressed genes
HPA: Human Protein Atlas
GTEx: Genotype-Tissue Expression
FANTOM5: Function Annotation of The Mammalian Genome
TCGA: The Cancer Genome Atlas
GEPIA2: Gene Expression Profiling Interactive Analysis
MGI: Mouse Genome Informatics
HE: hematoxylin-eosin
PTM: peritubular myoid cell
wpf: weeks postfertilization
ELISA: enzyme-linked immunosorbent assay
RT-qPCR: reverse transcription real-time polymerase chain reaction
WB: western blotting
VUS: unknown-significance variants
SO: severe oligospermia
FISH: fluorescence *in situ* hybridization
CCK-8: cell counting kit-8
TUNEL: TdT-mediated dUTP Nick-End Labeling
Co-IP: co-immunoprecipitation
RIP: RNA binding protein immunoprecipitation
DMR: differentially methylated region
PAS: periodic acid-schiff
WGBS: whole-genome bisulfite sequencing
ENA: European Nucleotide Archive
scATAC-seq: single-cell assay for transposase-accessible chromatin sequencing
OSCC: oral squamous cell carcinoma
TFs: transcription factors
hESC: human embryonic stem cells
PGCLC: primordial germ cell-like cells
TGCT: testicular germ cell tumor
ExAC: Exome Aggregation Consortium
CNV: copy number variation
EMT: epithelial-mesenchymal transition
ECM: extracellular matrix
